# Metabolic adaptation to IMMT deficiency through the ATF6-PPARγ axis is contingent on TP53 mutation status in breast cancer

**DOI:** 10.1038/s41419-026-08813-y

**Published:** 2026-04-28

**Authors:** Li Liu, Dan Li, Zeyu Hou, Yi Huang, Jinjing Wang, Chaorui Pu, Qingqing Zhao, Yunyan Yu, Rui Chen

**Affiliations:** 1 Department of General Surgery, Affiliated Hospital of Zunyi Medical University, Zunyi, Guizhou China; 2 Department of Thyroid and Breast Surgery, Affiliated Hospital of Zunyi Medical University, Zunyi, Guizhou China; 3 Department of General Surgery, The Second Affiliated Hospital of ZunYi Medical University, Zunyi, Guizhou China; 4 Department of Pathology, Affiliated Hospital of Zunyi Medical University, Zunyi, Guizhou China; 5 Department of Laboratory Medicine, Nanchuan District People’s Hospital, Chongqing, China

**Keywords:** Breast cancer, Endoplasmic reticulum, Stress signalling

## Abstract

Mitochondrial dysfunction and the corresponding metabolic reprogramming have been established as critical drivers of tumor progression; nevertheless, the specific molecular mechanisms have not yet been fully elucidated. In this study, we reveal that ablation of inner mitochondrial membrane protein (IMMT), a key architectural component of mitochondrial cristae, induces concurrent mitochondrial and endoplasmic reticulum stress (ERS), which selectively activates the ATF6-mediated unfolded protein response (UPR) to drive breast cancer (BC) cell proliferation. Mechanistically, IMMT loss promotes ATF6α–ATF6β heterodimer formation, whereby ATF6α stabilizes ATF6β protein, enabling ATF6β to engage PPARγ through direct physical interaction and orchestrate redox homeostasis remodeling that sustains tumor cell proliferation. Notably, we discovered that this compensatory stress adaptation is context-dependent, manifesting specifically in TP53-mutant tumors, but not in their wild-type counterparts, and targeted disruption of the ATF6β–PPARγ signaling axis effectively abrogates the oncogenic effects induced by IMMT-KO. Our work uncovers a previously unrecognized adaptive axis linking chronic mitochondrial dysfunction to redox control in BC and establishes ATF6β as a critical effector that partners with PPARγ under stress—a functional role distinct from its classical regulatory relationship with ATF6α. These findings provide a theoretical foundation for precision therapeutic strategies targeting vulnerabilities in the stress adaptation pathway of BC.

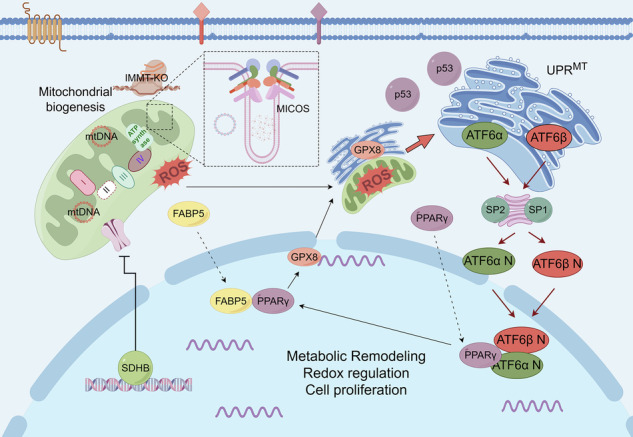

## Introduction

The tumor cells often reside in abnormal microenvironments characterized by nutrient deprivation, hypoxia, elevated metabolic demands, and oxidative stress, and thus must promptly adjust their state to facilitate survival [1]. This critical adaptation is primarily achieved through metabolic reprogramming. In this process, mitochondria—as the central organelles for cellular energy and material metabolism—facilitate tumor adaptation to pathological conditions and promote malignant progression [2, 3]. Earlier studies suggested that tumor cells preferentially utilize anaerobic glycolysis for energy production even under aerobic conditions, a phenomenon termed the Warburg effect [4]. However, emerging evidence indicates that cancer cells can flexibly switch between glycolysis and oxidative phosphorylation (OXPHOS) to fulfill the high energy demands associated with proliferation and growth [5, 6]. Beyond bioenergetics, tumor cells also exhibit metabolic adaptations in biomass synthesis, such as dependence on amino acids and enhanced lipogenesis, to supply the necessary building blocks for rapid proliferation [7, 8]. Given this metabolic plasticity and diversity, targeting mitochondrial metabolic reprogramming has emerged as a promising therapeutic strategy in cancer.

The endoplasmic reticulum (ER) is the primary site for protein synthesis and folding in eukaryotic cells [9]. During metabolic reprogramming, tumor cells can lead to the accumulation of misfolded proteins, thereby inducing endoplasmic reticulum stress (ERS) [10, 11]. When the accumulation of misfolded proteins exceeds the processing capacity of the ER, the cell activates the unfolded protein response (UPR) to alleviate ERS by modulating transcriptional and translational processes. Previous studies have demonstrated a functional interplay between mitochondria and the ER, which is fundamental for essential cellular functions [12, 13]. Notably, Mitochondria-Associated Endoplasmic Reticulum Membranes (MAMs) are specialized subdomains that mediate dynamic contacts between mitochondria and the ER, serving as critical platforms in tumor initiation and progression [14–16]. Although current research has established a pivotal role for MAMs in regulating tumor cell metabolic reprogramming, stress adaptation, and Ca²⁺ signaling, the underlying mechanisms are not yet fully elucidated.

IMMT, an inner mitochondrial membrane protein also known as Mic60 or Mitofilin [17], constitutes the core structural component of the mitochondrial contact site and cristae organizing system (MICOS) [18]. As a master regulator of mitochondrial inner membrane architecture and bioenergetics, IMMT orchestrates diverse cellular processes encompassing energy metabolism, autophagy regulation, and transmembrane signaling [19, 20]. Emerging evidence implicates IMMT dysregulation in the pathogenesis of cardiovascular diseases, neurodegenerative disorders, and infectious processes. Chaurembo et al. [21] demonstrated that genetic ablation of IMMT induces mitochondrial membrane ultrastructural abnormalities and bioenergetic deficits, culminating in myocardial ischemia-reperfusion injury. Complementary work by He et al. [22] revealed that IMMT impairment disrupts lipid homeostasis through destabilization of MAMs, thereby contributing to the development of neurodegenerative diseases. Current investigations of IMMT in oncological contexts remain predominantly restricted to bioinformatic analyses and clinicopathological correlations, with limited understanding of its specific mechanisms in tumor progression. While our prior investigations [23], corroborated by Lin et al. [24], established that acute siRNA knockdown of IMMT suppresses breast cancer (BC) proliferation by inducing severe mitochondrial oxidative stress and impairing OXPHOS, the long-term consequences of stable IMMT deficiency remained unexplored. Significantly, this condition better reflects the prolonged absence of IMMT observed in clinical tumor tissues due to adaptive survival. Meanwhile, the potential compensatory pathways that sustain tumor cell survival and growth following such alterations also require further investigation.

A prime candidate for mediating such adaptation is the ERS response, particularly the ATF6 branch, which is known to orchestrate metabolic reprogramming and lipid metabolism, partly through interactions with PPARα [25]. Moreover, the efficacy of this adaptive response is likely contingent on the genetic context. Among these, TP53 mutation is a key determinant of the cellular ERS response [26] and is the most frequent genetic alteration in BC [27].

Based on this background, we investigate the remodeling of energy metabolism and corresponding molecular mechanisms in IMMT-KO BC utilizing cell model along with clinical tissue specimens. Our results reveal a novel compensatory mechanism for mitochondrial dysfunction in which IMMT-KO activates the ATF6-mediated ERS response. This activation regulates metabolic reprogramming through the PPARγ–FABP5 signaling axis, significantly enhancing the proliferative capacity of BC cells in a manner dependent on the mutation status of the TP53 gene. These findings reveal promising therapeutic opportunities for targeting IMMT-deficient BC.

## Methods

### Cell lines and reagents

The cell lines in this study were sourced as follows: MDA-MB-231 and MCF-7 from the Cell Bank of Chinese Academy of Sciences (Shanghai, China); SK-BR-3, JIMT-1, and HCC-1954 from Procell Life Science & Technology Co., Ltd (Wuhan, China). All cells were cultured in supplier-recommended media containing 10% fetal bovine serum (FBS) and 1% penicillin-streptomycin (P/S), maintained at 37 °C under 5% CO₂ humidified atmosphere. Cell line authentication was performed using short tandem repeat (STR) profiling, with mycoplasma contamination excluded by Mycoplasma Detection Kit (Solarbio, Beijing, China). The detailed information regarding the cell used in this study is provided in Supplementary Table 1.

Ginsenoside Rh1, Ceapin-A7, and melatonin were procured from MedChemExpress (Monmouth Junction, NJ, USA). All compounds were dissolved in DMSO, aliquoted, and stored at −80 °C until use. Complete specifications of antibodies employed for western blotting are detailed in Supplementary Table 2.

### Clinical specimens

Fifty-eight paraffin-embedded tissue samples from patients with recurrent metastatic breast cancer were collected at the Affiliated Hospital of Zunyi Medical University between June 2015 and June 2024. Clinicopathological information and treatment courses were obtained from medical records, and patients with incomplete information were excluded. Informed consent was obtained from all participants. Informed consent was obtained from all participants. The study protocol was approved by the Ethics Committee of Zunyi Medical University (approval number: 2024-1-065). All procedures were conducted in accordance with the Helsinki Declaration. Patient clinical information is detailed in Supplementary Table 3.

### Bioinformatic analysis

Transcriptomic data from breast cancer patients were analyzed using The Cancer Genome Atlas (TCGA) (http://cancergenome.nih.gov/). Gene Set Enrichment Analysis (GSEA) was performed with the “ClusterProfiler” R package to investigate the functions of IMMT. The association between target gene expression and patient survival was analyzed using the Kaplan-Meier plotter (http://kmplotter.com), with calculation of the hazard ratio (HR), log-rank p-value, and 95% confidence interval (CI).

### siRNA transfection

#### In vitro experiments

ATF6α- and ATF6β-specific siRNA sequences (ATF6α-siRNA: 5′-GCACCCAAGACUCAAACAATT-3′ and 5′-UUGUUUGAGUCUUGGGUGCTT-3′; ATF6β-siRNA: 5′-CAGCAUUCUUGGAUGCAAUTT-3’ and 5’-AUUGCAUCCAAGAAUGCUGTT-3’) and scrambled negative control siRNA (5′-UUCUCCGAACGUGUCACGUTT-3′ and 5′-ACGUGACACGUUCGGAGAATT-3′), designed by Genepharma (Shanghai, China), were transfected using Lipofectamine 2000 (Thermo Fisher Scientific, USA) as the transfection reagent. When cell confluence reached 60–70%, siRNA and Lipofectamine 2000 were mixed in Opti-MEM (Gibco, USA) reduced serum medium, incubated at room temperature for 20 min, and then added to the cell culture system. After 8 h of transfection, the medium was replaced with complete medium, and cells were cultured for an additional 72 h. Knockdown efficiency was then verified by Western blot analysis of protein expression levels.

#### In vivo experiments

ESC-chemically modified ATF6α/β-specific siRNA (in vivo grade) from Genepharma (Shanghai, China) was used for in vivo gene silencing, with in vivo siRNA-mate (Shanghai, China) as the transfection reagent. When tumors reached a diameter of ~5 mm, mice were randomly assigned to one of three groups (*n* = 6/group) using a random number generator in Microsoft Excel. ESC-siRNA was dissolved in sterile saline and mixed with in vivo siRNA-mate at a 1:1 mass ratio. After vortexing and incubation at room temperature for 15 min to form complexes, the mixture was administered locally every other day, with saline-injected animals serving as controls (*n* = 6/group). Animals were sacrificed 48 h after the final administration, and target tissues were collected for analysis.

### Generation of IMMT-KO Cell Lines

The IMMT-KO cell lines were generated via the CRISPR/Cas9 system. Briefly, single-guide RNAs (sgRNAs: AATGGAACATTATAAGCTGC) targeting the IMMT gene were designed using the Broad Institute’s CRISPick online platform, with a non-targeting sgRNA serving as a negative control. The lentiviral IMMT knockout vector was constructed via ligation of hybridized oligonucleotides into the lentiCRISPRv2 puro plasmid (Addgene, USA) following established protocols [28]. Recombinant plasmids were co-transfected with packaging plasmids (4 μg psPAX2 [Addgene, USA] and 2 μg pMD2.G [Addgene, USA]) into Lenti-X 293T cells using the Lipofectamine 3000 transfection reagent (Thermo Fisher Scientific, USA) for lentiviral production. Culture medium was replaced 6 h post-transfection, and lentivirus-containing supernatant was harvested from 293T cells 48 h after transfection. Lentiviral supernatant containing 8 μg/mL polybrene (Beyotime Biotechnology, China) was utilized for cell transduction, after which puromycin selection (1 μg/mL; Beyotime Biotechnology, China) was performed for 7 days. Surviving monoclonal populations were expanded, and successful IMMT knockout was verified via western blotting.

### Proteome analyses

Proteomic analysis was performed by APTBIO Company (Shanghai, China). Cell pellets (5 × 10⁶ cells per sample, *n* = 5 per group) were collected, snap-frozen in liquid nitrogen, and shipped on dry ice. Proteins were extracted using SDT lysis buffer (4% SDS, 100 mM Tris-HCl, pH 7.6) and quantified via the BCA assay. After tryptic digestion via the filter-aided sample preparation (FASP) method, peptide mixtures were desalted and reconstituted. Data-independent acquisition (DIA) analysis was performed on an Orbitrap Astral mass spectrometer (Thermo Scientific) coupled to a nanoflow Vanquish Neo UHPLC system (Thermo Scientific). Quality Control and Data Processing: To monitor system stability, a pooled quality control (QC) sample was analyzed at regular intervals throughout the LC-MS/MS run. Data reproducibility was evaluated by calculating the median coefficient of variation (CV) across all QC samples, which was <20%, indicating a stable analytical platform. DIA data were processed using DIA-NN software (version 1.8) with the following parameters: enzyme, trypsin (max missed cleavages = 1); fixed modification, carbamidomethylation (C); variable modifications, oxidation (M) and N-terminal acetylation; and false discovery rate (FDR) threshold for protein identification, <1%. Differentially expressed proteins (DEPs) were defined as those with |fold change| > 1.5 and adjusted p-value (FDR) < 0.05. The Gene Ontology (GO) functional annotation of all differentially expressed proteins (DEPs) was analyzed using the Blast2GO software (version BLASTP 2.8.0+). In addition, the KOBAS software (version 3.0) was used to perform KEGG pathway annotation on the target protein set.

### Metabolomic profiling

Untargeted metabolomics analysis was performed by Wuhan MetWare Biotechnology Co., Ltd. Cell pellets (5 × 10⁶, *n* = 5 per condition) were snap-frozen and shipped for analysis. The workflow comprised two stages: (1) Untargeted profiling on a Q-TOF mass spectrometer (SCIEX) using a UPLC HSS T3 column (positive/negative ion mode) and a BEH HILIC column (negative ion mode). (2) Targeted quantification on an ESI-QTRAP mass spectrometer (QTRAP® LC-MS/MS System, SCIEX) in MRM mode. Quality control (QC) samples were analyzed throughout the run to ensure data reproducibility. Differential metabolites were identified with thresholds of variable importance in projection (VIP) > 1.0 and *p*-value < 0.05 (Student’s *t* test).

### Transmission electron microscopy sample preparation and observation

The preparation and observation of TEM samples in this study were performed by Wuhan Microspectra Biotechnology Co. (Wuhan, China), Ltd. The specific procedures were as follows: samples were fixed with 2.5% glutaraldehyde at room temperature for 2 h, followed by three washes with PBS, and then post-fixed with osmium tetroxide solution at 4 °C in the dark for 2 h. After three washes with deionized water (ddH₂O), the samples were dehydrated through a graded ethanol series (30%, 50%, 70%, 90%, and 100%), transitioned with propylene oxide, and embedded after gradient infiltration with 812 resin. The embedded blocks were sectioned into ultrathin slices using a Leica UC7 ultramicrotome, double-stained with uranyl acetate and lead citrate, and finally observed under a Hitachi HT7800 (Japan) transmission electron microscope to examine the cellular ultrastructure.

### Western blot assays

After cell collection, total protein was extracted by lysing cells with RIPA lysis buffer containing 1 mM PMSF (Solarbio, China) and protease and phosphatase inhibitor cocktail (100×) (Solarbio, China). Protein concentration was determined using the BCA Protein Assay Kit (Thermo Fisher Scientific, USA). Equal amounts of protein samples were separated by 10% SDS-PAGE and transferred onto PVDF membranes (Merck Millipore, Germany). After transfer, the membranes were blocked with 2% BSA (Solarbio, China) in TBST buffer at room temperature for 1 h. The membranes were then incubated with primary antibodies diluted according to the manufacturer’s recommendations at 4 °C overnight. The next day, after washing with TBST, the membranes were incubated with corresponding secondary antibodies at room temperature for 2 h. Finally, target proteins were detected using an ECL chemiluminescence detection system, and the band intensities were quantified using ImageJ software.

### Immunoprecipitation

immunoprecipitation (IP) was performed using the Thermo Fisher Pierce™ Classic IP Kit (Thermo Fisher Scientific, USA). First, cells were collected and washed twice with ice-cold PBS, followed by lysis in the kit-provided lysis buffer on ice for 10 min. After lysis, the samples were centrifuged at 12,000 × *g* for 10 min at 4 °C, and the supernatant was collected. Protein concentration was determined using the BCA assay. A total of 500 μg of protein was incubated with 10 μg of the target protein antibody overnight at 4 °C with rotation. The next day, 40 μL of pre-washed Protein A/G agarose beads was added, and the mixture was incubated at 4 °C with rotation for 1 h. After incubation, the samples were centrifuged at 1000 × *g* for 1 min at 4 °C, and the supernatant was discarded. The beads were washed three times with ice-cold lysis buffer. Finally, 2× SDS loading buffer containing DTT (final concentration: 20 mM) was added, and the samples were boiled for 10 min, followed by centrifugation for subsequent Western blot analysis.

### Immunofluorescence (IF) staining

Cells were seeded in confocal dishes and fixed with 4% paraformaldehyde (Solarbio, China) at room temperature for 15 min. After fixation, the cells were washed three times with PBS, for 5 min each. Subsequently, the cells were permeabilized with 0.5% Triton X-100 (Solarbio, China) at room temperature for 10 min. After permeabilization, the cells were blocked with 10% BSA in PBS at room temperature for 1 h. Primary antibodies, diluted in blocking buffer according to the manufacturer’s recommendations, were applied and incubated at 4 °C overnight. The next day, the cells were washed three times with PBS, 5 min each, followed by incubation with fluorescence-labeled secondary antibodies (protected from light) at room temperature for 1 h. Finally, the cells were washed three times with PBS, 5 min each, and nuclei were stained with DAPI (Solarbio, China) for 5 min. The samples were observed and imaged using a laser scanning confocal microscope (ZEISS, Germany) with consistent acquisition settings.

### Detection of mitochondrial reactive oxygen species and membrane potential

After seeding the test cells into confocal-specific culture dishes and allowing them to adhere for 24 h, independent detections were performed as follows: the mitochondrial superoxide detection group was incubated with HBSS buffer containing 5 μM Mito-SOX™ Red (Thermo Fisher Scientific, USA) in the dark for 30 min (37 °C), while the mitochondrial membrane potential detection group was incubated with HBSS buffer containing 5 μM Image-iT™ TMRM (Thermo Fisher Scientific, USA) under the same conditions. Both groups were washed three times with pre-warmed HBSS to remove unbound probes. Following staining, imaging was performed using a laser confocal microscope with excitation/emission wavelengths set to 510/580 nm for Mito-SOX™ Red and 543/576 nm for TMRM. Five random fields of view were selected for each sample, and the average fluorescence intensity was quantified using ImageJ software. All experimental groups maintained the same exposure parameters.

### Proximity ligation assays

Protein-protein interactions were detected using the Duolink® PLA kit (Sigma-Aldrich, GER). Briefly, cells grown on confocal dishes were fixed, permeabilized, and blocked with the provided blocking solution. Primary antibodies against the target proteins were incubated overnight at 4 °C, followed by species-specific Duolink secondary antibodies. The PLA signal was developed through ligation (30 min, 37 °C), polymerase-mediated amplification (100 min, 37 °C), and fluorescence detection. Nuclei were counterstained with DAPI for spatial reference. Quantitative analysis of Proximity ligation assays (PLA) signals: All images were acquired using a confocal microscope with consistent acquisition parameters. For quantitative analysis, three non-overlapping fields of view were randomly selected from each of the three independent biological replicates. Within each field, all cells exhibiting intact nuclear morphology and clear signals were included. This sampling strategy ensured that a minimum of 15 cells were cumulatively analyzed per experimental condition. PLA foci were identified according to stringent criteria: only structures displaying clearly delineated punctate morphology and fluorescence intensity markedly above the local background were defined as an individual focus. All foci were manually identified and counted by an analyst blinded to the experimental group assignments. The mean number of foci per cell was used as the quantitative measure. Data were visualized, and the means with standard errors were calculated and subjected to appropriate significance tests using GraphPad Prism.

### Mitochondrial isolation

Mitochondrial isolation was performed using the Abcam Mitochondria Isolation Kit (Abcam, UK). Briefly, approximately 1 × 10⁷ cells were collected, washed twice with pre-cooled PBS, and resuspended in 1 mL of Reagent A. After incubation on ice for 15 min, the cell suspension was transferred to a pre-cooled Dounce grinder and subjected to 40 strokes to mechanically disrupt the cell membranes while maintaining 4 °C. The lysate was transferred to a 1.5 mL centrifuge tube and centrifuged at 1000 × *g* for 10 min at 4 °C. The supernatant was collected into a new tube, while the cell was resuspended in 1 mL of Reagent B and grinder again. The resulting supernatant was combined with the first supernatant, and the mixture was centrifuged at 12,000 × *g* for 15 min at 4 °C. The supernatant was discarded, and the mitochondrial was collected. The mitochondrial protein concentration was determined using the BCA assay.

### Cytosolic and nuclear fractionation

According to the instructions of Thermo Fisher Scientific’s NE-PER™ Nuclear and Cytoplasmic Extraction Reagents (Thermo Fisher Scientific, USA), the brief procedure is as follows: First, collect the cells and wash them with PBS, then centrifuge to obtain the cell. Resuspend the cell in ice-cold Cytoplasmic Extraction Reagent I, vortex to mix thoroughly, and incubate on ice. Next, add Cytoplasmic Extraction Reagent II, vortex to mix, and centrifuge to collect the supernatant, which is the cytoplasmic fraction. Then, resuspend the nuclear in ice-cold Nuclear Extraction Reagent, vortex to mix, and incubate on ice with periodic vortex. Finally, centrifuge to collect the supernatant, which is the nuclear fraction. Both fractions can be used for subsequent Western blot analysis.

### Animal experiments

All animal experiments were performed using nude mice obtained from the Public Experimental Animal Center of Zunyi Medical University. Tumor cells in the logarithmic growth phase were resuspended in sterile PBS and mixed with 10% Matrigel to ensure proper cell suspension and tumor formation. Four-week-old female BALB/c nude mice were injected subcutaneously in the right axilla with 100 μL of the cell suspension. After injection, the general condition of the mice, including activity, diet, and injection site reactions, was monitored daily. Tumor length (*L*) and width (*W*) were measured every 3 days using a caliper, and tumor volume was calculated using the formula *V* = (*L* × *W*²)/2. When the tumor diameter reached approximately 2 cm, the mice were euthanized by cervical dislocation, and the tumor tissues were completely excised and weighed. All animal experiments were conducted in compliance with the Zunyi Medical University Guidelines for Animal Research and Use (Approval No. zyfy-an-2024-0006). The mice were housed in an SPF-grade environment under a 12 h light/dark cycle with free access to food and water. All animals that received tumor cell inoculation and survived the full treatment course were included in the analysis. No animals were excluded post-hoc based on outcome. All measurements were based on objective criteria and automated analysis to reduce observer bias.

### Immunohistochemical assays and scoring

To detect the expression of IMMT, Ki-67, ATF6α, ATF6β, PPARγ, FABP5 and GPX8 in xenografted tumor samples, we performed immunohistochemical (IHC) staining. A scoring system ranging from 0 to 12 was used to evaluate the staining results, which was obtained by multiplying the signal intensity score with the percentage score of positive staining cells. The signal intensity was scored as follows: 0 (no signal), 1 (weak), 2 (moderate), and 3 (strong). The percentage of stained cells was scored as: 1 (<10%), 2 (10%–49%), 3 (50%–74%), and 4 (>75%). Six tumor sections were analyzed per group, and three randomly selected fields were counted from each section. The percentage of Ki-67 positive cells was calculated by dividing the number of positive cells by the total number of cells.

### CCK8, clone formation and EDU Assay

Cells in the logarithmic growth phase were seeded into a 96-well plate at a density of 3000 cells per well (100 μL per well) and cultured in a 37 °C incubator with 5% CO₂. The time points for measurement were set at 24, 48, 72, and 96 h. At each time point, 10 μL of CCK8 reagent was added to each well, and the plate was incubated at 37 °C for another 2 h. Then, the absorbance value of each well was measured at 450 nm using a microplate reader, with five replicate wells set for each group. Meanwhile, cells were seeded into 6-well and 12-well plates at an appropriate density and cultured in a 37 °C incubator with 5% CO₂ for 1 week. Different concentrations of drug solutions were then added to the plates, with at least three replicate wells set for each concentration. After 48 h of drug treatment, the culture medium was replaced, and the cells were cultured for another week. The supernatant was discarded, and the cells were washed carefully with PBS twice, fixed with 4% paraformaldehyde for 15 min, and then stained with crystal violet staining solution for 10 min. For the EdU assay, using the BeyoClick™ EdU-594 Cell Proliferation Kit (Beyotime, China) according to the manufacturer’s protocol.

### Measurement of oxygen consumption rate and extracellular acidification rate

The oxygen consumption rate (OCR) and extracellular acidification rate (ECAR) were measured using the Seahorse XFe96 Analyzer (Agilent, USA). Briefly, cells were seeded at a density of 3 × 10⁴ cells per well in a Seahorse XFe96 cell culture plate and cultured for 24 h to allow adherence. One hour before the assay, the growth medium was replaced with serum-free DMEM lacking sodium bicarbonate. For OCR measurement, the medium was supplemented with 10 mM glucose, 1 mM sodium pyruvate, and 2 mM glutamine, while for ECAR measurement, the medium was supplemented with 2 mM glutamine (both without sodium bicarbonate). The cells were then equilibrated in a 37 °C non-CO₂ incubator for 45 min. After calibrating the sensor cartridge, mitochondrial stress test reagents (1.5 μM oligomycin, 1 μM FCCP, and 0.5 μM rotenone/antimycin A) or glycolysis stress test reagents (10 mM glucose, 1 μM oligomycin, and 50 mM 2-DG) were sequentially injected. Each measurement cycle consisted of 3 min of mixing, 2 min of waiting, and 3 min of data acquisition. Data were analyzed using Wave software (Agilent, USA), with OCR expressed as oxygen consumption rate (pmol/min/cells) and ECAR as proton efflux rate (pmol/min/cells), normalized to baseline values.

### Glutathione (GSH/GSSG) content

The GSH and GSSG Assay Kit (Beyotime, China) was used to measure the levels of reduced glutathione (GSH) and oxidized glutathione (GSSG) in cells according to the manufacturer’s protocol.

### NADP+/NADPH

The NADP+/NADPH Assay Kit (Beyotime, China) was used to quantify cellular levels of oxidized (NADP+) and reduced (NADPH) nicotinamide adenine dinucleotide phosphate following the manufacturer’s standardized protocol.

### L-Lactic acid content

The L-lactic acid content was quantified using the L-Lactic Acid Assay Kit (Njjcbio, China) according to the manufacturer’s instructions. The lactic acid concentration was normalized to the total protein content determined by the BCA method (unit: mmol/mg protein).

### Mitochondrial and endoplasmic reticulum morphology analysis

Live-cell double staining was performed as follows: MitoTracker™ Green FM (Beyotime, China) mitochondrial green fluorescent probeand ER-Tracker™ Red (Thermo Fisher Scientific, USA) ER red fluorescent probe were diluted to a final concentration of 5 μM in serum-free medium and pre-warmed at 37 °C for 10 min. The original medium in the cell culture dish was aspirated, and cells were gently washed twice with pre-warmed HBSS. The medium containing the dual-staining probes was then added, followed by incubation at 37 °C in the dark for 30 min. After incubation, cells were washed three times with HBSS, and images were acquired under a confocal microscope.

### Statistical analyses

Statistical analyses were performed using GraphPad Prism (version 9.0). Unless otherwise specified in the figure legends, all data are derived from at least three independent biological replicates and are presented as mean ± standard deviation. For comparisons between two groups, two-tailed unpaired Student’s t-tests were applied. For comparisons among more than two groups, one‑way analysis of variance (ANOVA) followed by Tukey’s post‑hoc test was used. A *p*‑value < 0.05 was considered statistically significant. Graphical abstract by *Figdraw*.

## Result

### IMMT-KO promotes BC cell proliferation both in vitro and in vivo

**Fig. 1:**
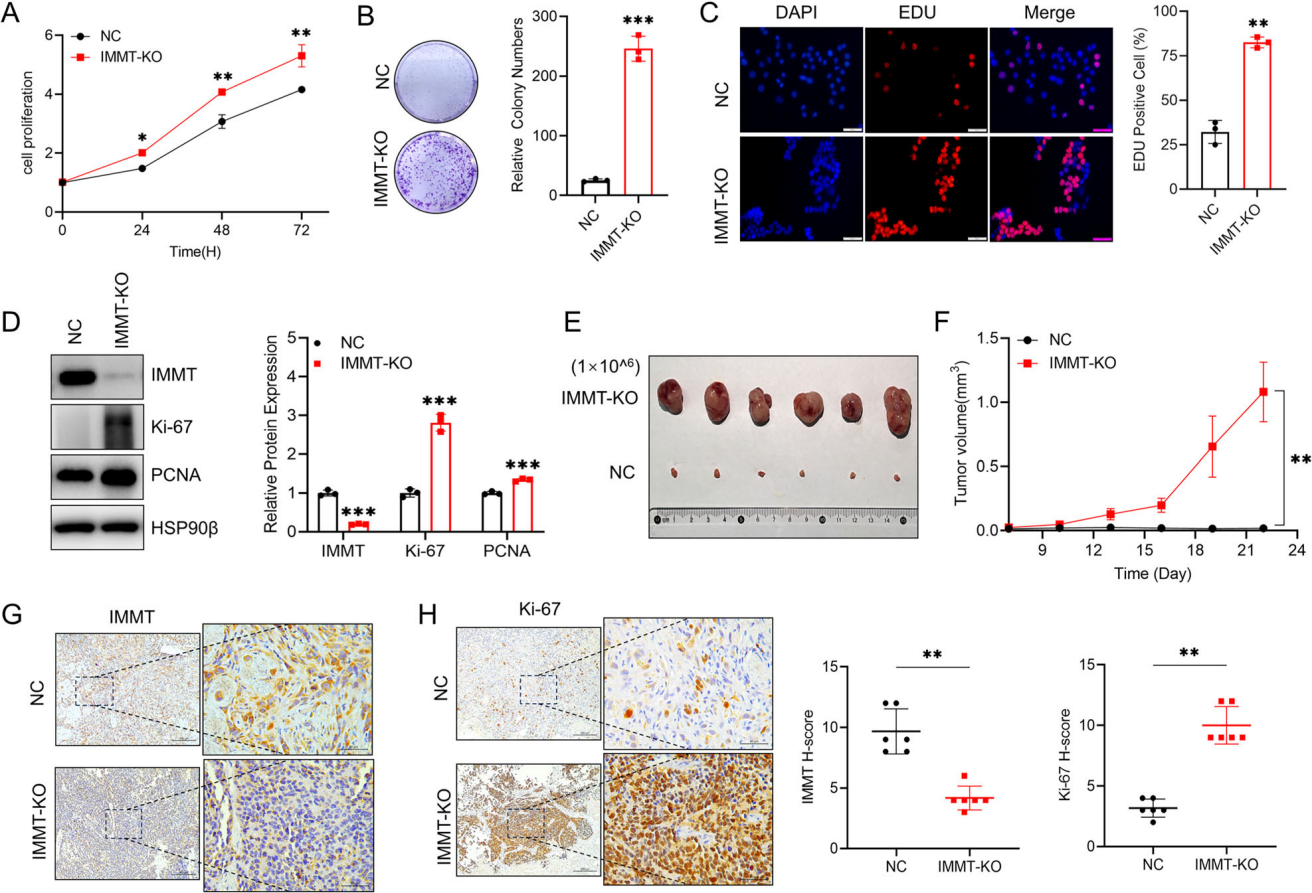
IMMT deficiency promotes SK-BR-3 cell proliferation and accelerates tumor growth in vivo. **A** CCK-8 assay was performed to evaluate the proliferative activity of SK-BR-3 cells following IMMT-KO (*n* = 5). **B** Colony formation assay was conducted to quantify the clonogenic ability of SK-BR-3 cells (*n* = 3). **C** EdU staining was employed to assess DNA synthesis activity in SK-BR-3 cells. **D** Western blot analysis was conducted to examine the protein expression of proliferation markers Ki-67 and PCNA. **E** Representative images of tumors isolated from tumor-bearing mice (*n* = 6). **F** Tumor growth curves of these mice over time. **G**, **H** IHC analysis was performed to assess the expression of IMMT and Ki-67 in tumor tissues. Data are presented as mean ± SD. Statistical significance: *P < 0.05, **P < 0.01, ***P < 0.001.

In our previous study, we demonstrated that transient knockdown of IMMT inhibited the proliferative capacity of BC cells by affecting mitochondrial morphology and function [23]. To investigate the associated mechanisms, we established an IMMT-KO SK-BR-3 cell using CRISPR-Cas9. Intriguingly, contrary to the transient knockdown phenotype, IMMT-KO cells exhibited enhanced proliferative capacity, as evidenced by CCK-8 (Fig. 1A), colony formation (Fig. 1B), and EdU incorporation assays (Fig. 1C). Western blot analysis further revealed significant upregulation of proliferation markers Ki-67 and PCNA in IMMT-KO cells (Fig. 1D).

To validate these findings in vivo, 1 × 10⁶ control or IMMT-KO SK-BR-3 cells were subcutaneously injected into 4-week-old female BALB/c nude mice (*n* = 6/group). Xenograft analysis revealed significantly increase in tumor volume at day 22 post-implantation in the knockout group (Fig. 1E, F). Correspondingly, IHC analysis of xenografts confirmed successful IMMT-KO, and further demonstrated dramatically increase in Ki-67-positive cells in IMMT-KO tumors (Fig. 1G, H). These results suggest that IMMT-KO may activate compensatory oncogenic pathways distinct from acute mitochondrial dysfunction induced by transient knockdown.

### Stable IMMT-KO remodels redox homeostasis in BC cells

**Fig. 2:**
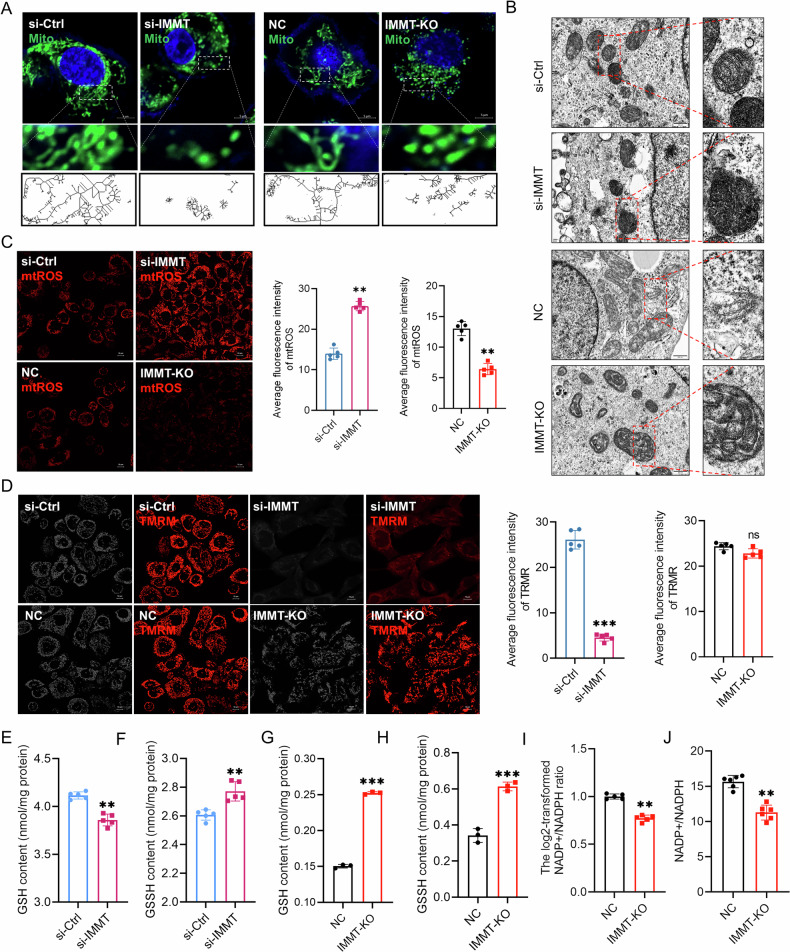
Effects of IMMT deficiency on mitochondrial function and redox homeostasis. **A** Mitochondrial morphology in SK-BR-3 cells following IMMT knockdown or knockout was observed using Mito-Tracker™ Green staining. **B** Mitochondrial ultrastructure was examined using TEM. **C** Mitochondrial superoxide levels were measured by Mito-SOX™ Red fluorescence staining. **D** Mitochondrial membrane potential (Δ*Ψ*m) was evaluated using TMRM fluorescence staining. **E**–**H** Cellular glutathione levels. **I**, **J** Cellular NADP + /NADPH ratio. Data represent mean ± SD. Statistical significance: **P < 0.01, ***P < 0.001.

Given the central role of IMMT in mitochondrial homeostasis, we conducted comparative analyses of mitochondrial structure and function in the SK-BR-3 cell between transient knockdown and stable knockout models. Mito-Tracker staining demonstrated that both experimental groups induced mitochondrial fragmentation, manifested as a shift from filamentous to punctate morphology (Fig. 2A). Through transmission electron microscopy analysis, control cells were observed to maintain mitochondria with well-defined cristae, while both IMMT knockdown and IMMT-KO cells showed cristae loss accompanied by distinct “onion-like” membrane structures (Fig. 2B), indicating profound inner membrane remodeling. Notably, the transient knockdown model showed significantly increased mtROS levels concomitant with diminished membrane potential (Δ*Ψ*m), suggesting acute oxidative stress and functional impairment (Fig. 2C, D). Conversely, stable knockout cells displayed lower mtROS levels relative to controls with preserved Δ*Ψ*m, indicating cellular adaptation to chronic IMMT deficiency via attenuated oxidative stress responses. Acute IMMT knockdown by siRNA resulted in a significant decrease in GSH and a concomitant increase in GSSG, consistent with acute mitochondrial dysfunction and oxidative stress. In stark contrast, stable IMMT-KO cells exhibited a compensatory redox reprogramming, characterized by concurrent elevation of both GSH and GSSG (Figs. 2E, F and S1A). Significantly, the NADP+/NADPH ratio was markedly reduced (Fig. 2G, H), indicating enhanced NADPH availability and reinforced cellular reducing capacity. These findings demonstrate that IMMT-KO induces compensatory metabolic reprogramming in BC cells to establish a fortified redox balance, in contrast to the acute oxidative disruption observed in transient knockdown.

### IMMT deficiency rewires metabolic flux to TCA cycle with compromised OXPHOS assembly

**Fig. 3:**
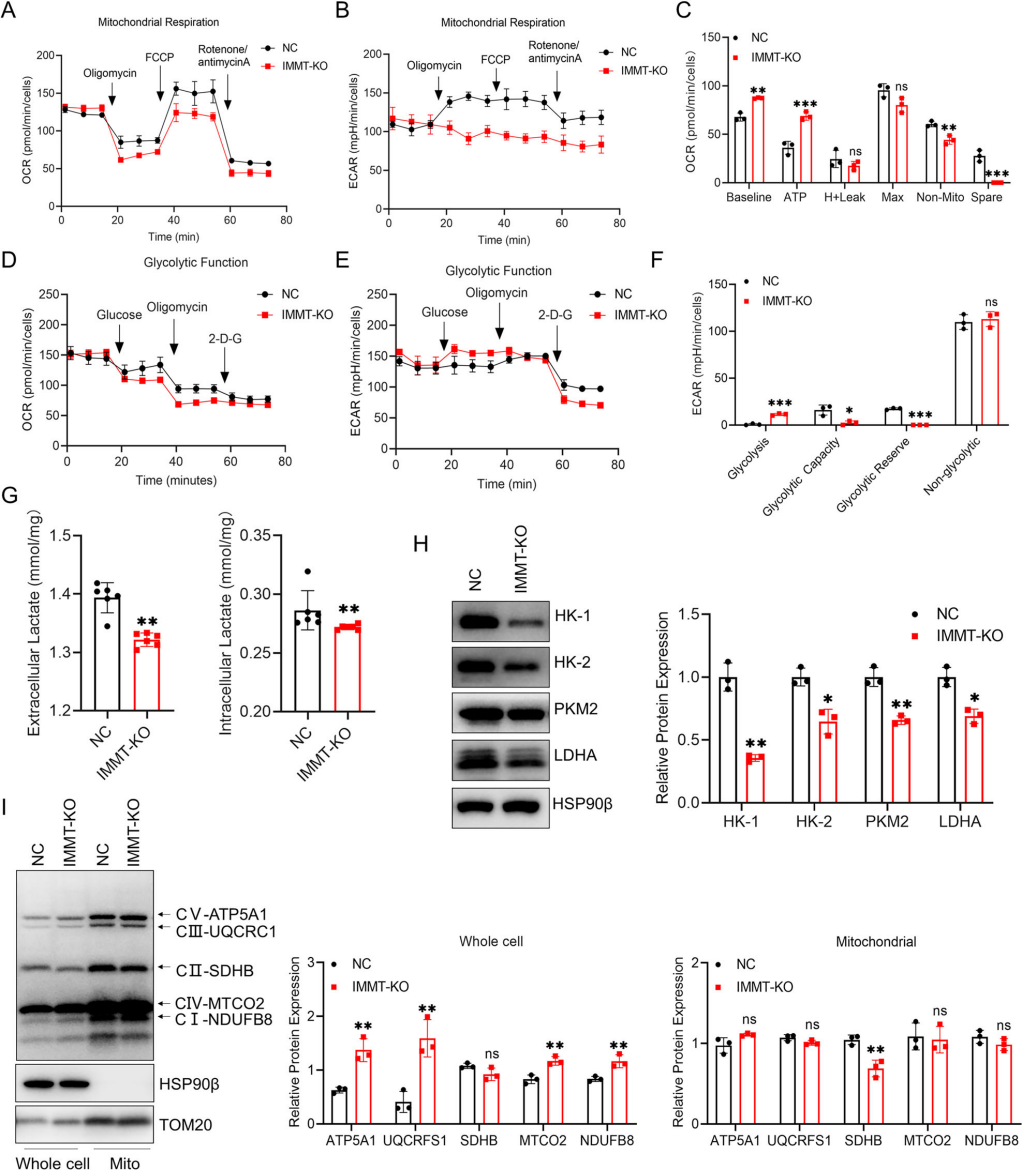
IMMT deficiency affects mitochondrial respiration and glycolytic capacity. **A**–**C** Mitochondrial respiratory function in IMMT-KO cells were assessed using a Seahorse XF Analyzer. **D**–**F** Glycolytic capacity was evaluated in IMMT-KO cells. **G** Intra- and extracellular lactate concentrations were determined in IMMT-KO cells. **H** Expression levels of key glycolytic enzymes HK1, HK2, PKM2, and LDHA were determined by western blotting. **I** Mitochondrial electron transport chain complexes CI–CV were detected by western blot. Data represent mean ± SD. Statistical significance: *P < 0.05, **P < 0.01, ***P < 0.001.

To investigate the impact of IMMT deficiency on cellular energy metabolism, we utilized the Seahorse XFe96 Analyzer to assess glycolysis and mitochondrial respiration in stable knockout cells compared to controls. Mitochondrial stress tests revealed that IMMT-KO cells exhibited a slight increase in basal respiration (Fig. 3A) and enhanced ATP production capacity, but was accompanied by a significant reduction in non-mitochondrial respiration and a severely impaired spare respiratory capacity (Fig. 3C). Notably, the knockout group did not show a significant increase in ECAR following oligomycin treatment (Fig. 3B), suggesting limited glycolytic compensation and an inability to effectively respond to OXPHOS inhibition. Additionally, after treatment with rotenone and antimycin A, ECAR decreased slightly in both groups, indicating that the increase in ECAR was partially derived from citric acid cycle (TCA) activity rather than glycolysis alone. ECAR stress tests further revealed that under glucose-free conditions, the OCR of IMMT-KO cells was slightly higher than that of controls (Fig. 3D). Moreover, both glycolytic capacity and reserve were significantly reduced in the knockout group compared to controls (Fig. 3F). Upon glucose addition, ECAR in the knockout group increased markedly, while OCR in the control group rose significantly, indicating that IMMT-KO cells rely more on the TCA cycle than glycolysis for ATP generation. This metabolic profile is consistent with the observed decrease in ECAR and persistently lower OCR in the knockout group after oligomycin treatment, highlighting significant mitochondrial functional vulnerability (Fig. 3E). To clarify the metabolic source of the elevated ECAR, we measured intracellular and extracellular lactate levels. The results showed that both intracellular and extracellular lactate levels were reduced in the IMMT-KO group compared to the control group (Fig. 3G). Combined with the significant downregulation of key glycolytic enzymes (Fig. 3H), we confirmed that glycolytic activity was markedly suppressed in the knockout group. Since CO₂ produced by the TCA cycle also contributes to ECAR through carbonic anhydrase-mediated acidification [29, 30], these results indicate that the increase in ECAR in the knockout group primarily stems from enhanced TCA cycle activity rather than lactate secretion from glycolysis.

Mitochondrial proteins were isolated and subjected to Western blot analysis to assess the impact of IMMT-KO on OXPHOS complexes. (Fig. 3I). Interestingly, total protein analysis showed upregulation of complexes I, III, IV, and V, while isolated mitochondrial proteins revealed a significant reduction only in complex II, with no notable changes in other complexes. This contradictory result suggests that IMMT-KO may interfere with the proper assembly or mitochondrial localization of these complexes through an unknown mechanism, rather than directly suppressing their expression. These findings indicate that, following stable IMMT-KO, cells partially restore mitochondrial function through metabolic reprogramming. However, limited glycolytic compensation and abnormal OXPHOS complex assembly result in persistent mitochondrial functional vulnerability. In summary, the experimental results presented above illustrate that cells, upon IMMT-KO, undergo a compensatory metabolic adaptation program.

### PPARγ-FABP5 axis is activated to maintain redox homeostasis and enhance proliferation in IMMT-KO cells

**Fig. 4:**
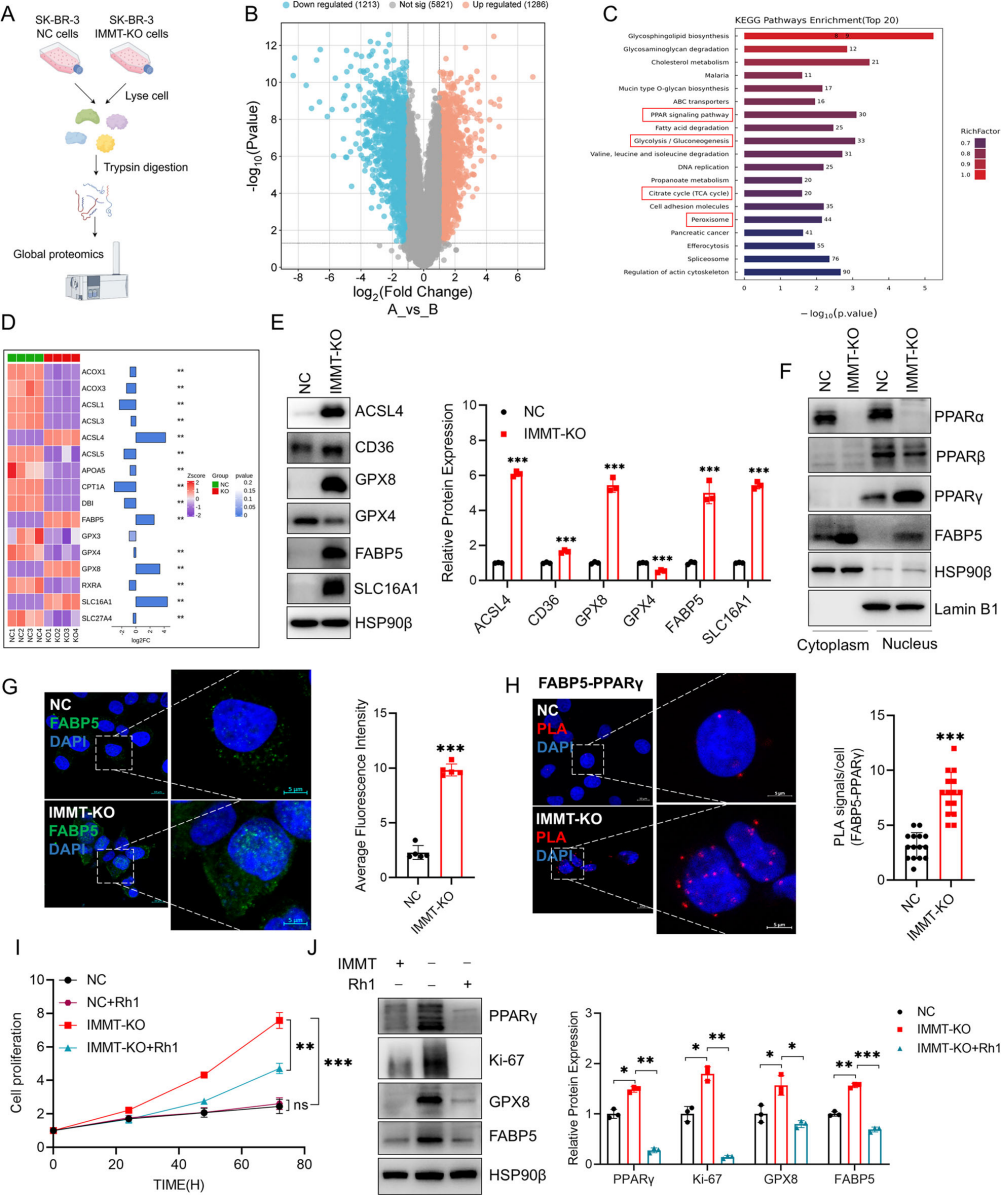
IMMT deficiency activates PPARγ-FABP5 signaling axis to drive metabolic adaptation. **A** Schematic of the global proteomic analysis workflow for IMMT-KO (By *Figdraw*). **B** Volcano plot displaying differentially expressed proteins. **C** Top 20 significantly enriched KEGG pathways identified by whole-cell proteomics in IMMT-KO cells. **D** Heatmap visualization of core PPAR signaling pathway components. **E** Western blot verification of PPAR pathway-associated proteins. **F** Subcellular distribution analysis of PPAR isoforms and FABP5 in cytoplasmic and nuclear fractions by western blotting. **G** IF staining for FABP5 to determine its subcellular localization. **H** PLA validating physical interaction between FABP5 and PPARγ. **I** Cell proliferation assessed by CCK-8 assay following Rh1 treatment (10 μM, 24 h) (*n* = 5). **J** Western blot analysis of PPARγ, Ki-67, FABP5, and GPX8 expression in Rh1-treated cells (10 μM, 24 h). Data represent mean ± SD. Statistical significance: *P < 0.05, **P < 0.01, ***P < 0.001.

To elucidate the molecular mechanisms underlying the metabolic phenotype transformation induced by IMMT-KO, we performed quantitative proteomic analysis (Fig. 4A). Volcano plot analysis demonstrated differential expression profiles between groups, with IMMT-KO cells exhibiting 1286 upregulated proteins and 1213 downregulated proteins compared to controls (Fig. 4B). KEGG pathway analysis revealed significant enrichment of key metabolic processes including cholesterol metabolism, PPAR signaling, peroxisome function, glycolysis/gluconeogenesis, and TCA cycle regulation (Fig. 4C). Topological analysis of the five most enriched pathways using chord diagrams identified PPAR signaling as the central regulatory node (Fig. S1B), a finding of particular significance given its established role in coordinating antioxidant responses and mitochondrial functional homeostasis [31–33]. To validate the above findings, proteomic heatmap analysis revealed significantly upregulated expression of key molecules in the PPAR signaling pathway including ACSL4, FABP5, GPX8, and SLC16A1, were specifically upregulated in the knockout group (Fig. 4D), and these results were further confirmed by Western blot (Fig. 4E).

Subsequently, we examined changes in the three key PPAR protein family subunits—PPARα, PPARβ, and PPARγ—before and after stable IMMT-KO. Nuclear-cytoplasmic fractionation experiments revealed that nuclear expression of PPARγ was significantly upregulated in IMMT-KO cells (Fig. 4F), while PPARα and PPARβ expression decreased. Research indicates that the PPAR family interacts with FABP5 within the nucleus, and this process serves as a key mechanism regulating fatty acid transport and metabolism [34]. Experimental results showed that nuclear translocation of FABP5 was significantly increased in the knockout group (Fig. 4F), which was further validated by IF localization (Fig. 4G). To determine whether PPARγ and FABP5 directly interact, we performed PLA. The results demonstrated a significant increase in PPARγ-FABP5 complex formation following IMMT-KO (Fig. 4H), suggesting their cooperative regulation of downstream gene expression. Untargeted metabolomics revealed that IMMT deficiency led to a significant remodeling of the global metabolic landscape (Fig. S1C). Notably, we observed specific alterations in fatty acid and phospholipid profiles, which are closely linked to PPARγ signaling (Fig. S1D). These metabolic changes provide functional validation for the activation of the PPARγ-FABP5 axis, suggesting that the ensuing lipid metabolic reprogramming may supply both biosynthetic substrates and energy to fuel the accelerated proliferation of cancer cells.

To elucidate the central regulatory role of PPARγ in metabolic reprogramming and antioxidant response following IMMT-KO, we performed functional rescue experiments using the PPARγ-specific inhibitor Ginsenoside Rh1. The CCK8 assay results demonstrate that Rh1 treatment significantly inhibited the proliferation of IMMT-KO cells (Fig. 4I), while the Western blot results indicate that the expression of FABP5/GPX8 was significantly suppressed (Fig. 4J). Further examinations revealed that Rh1 treatment induced mtROS accumulation and significant mitochondrial membrane depolarization (Fig. S1E, F). These findings indicate that IMMT-KO selectively activates the PPARγ-FABP5 axis, driving metabolic reprogramming and antioxidant stress responses to support adaptive cell proliferation.

### IMMT deficiency coordinates Mitochondria-ER stress signaling through ATF6 activation

**Fig. 5:**
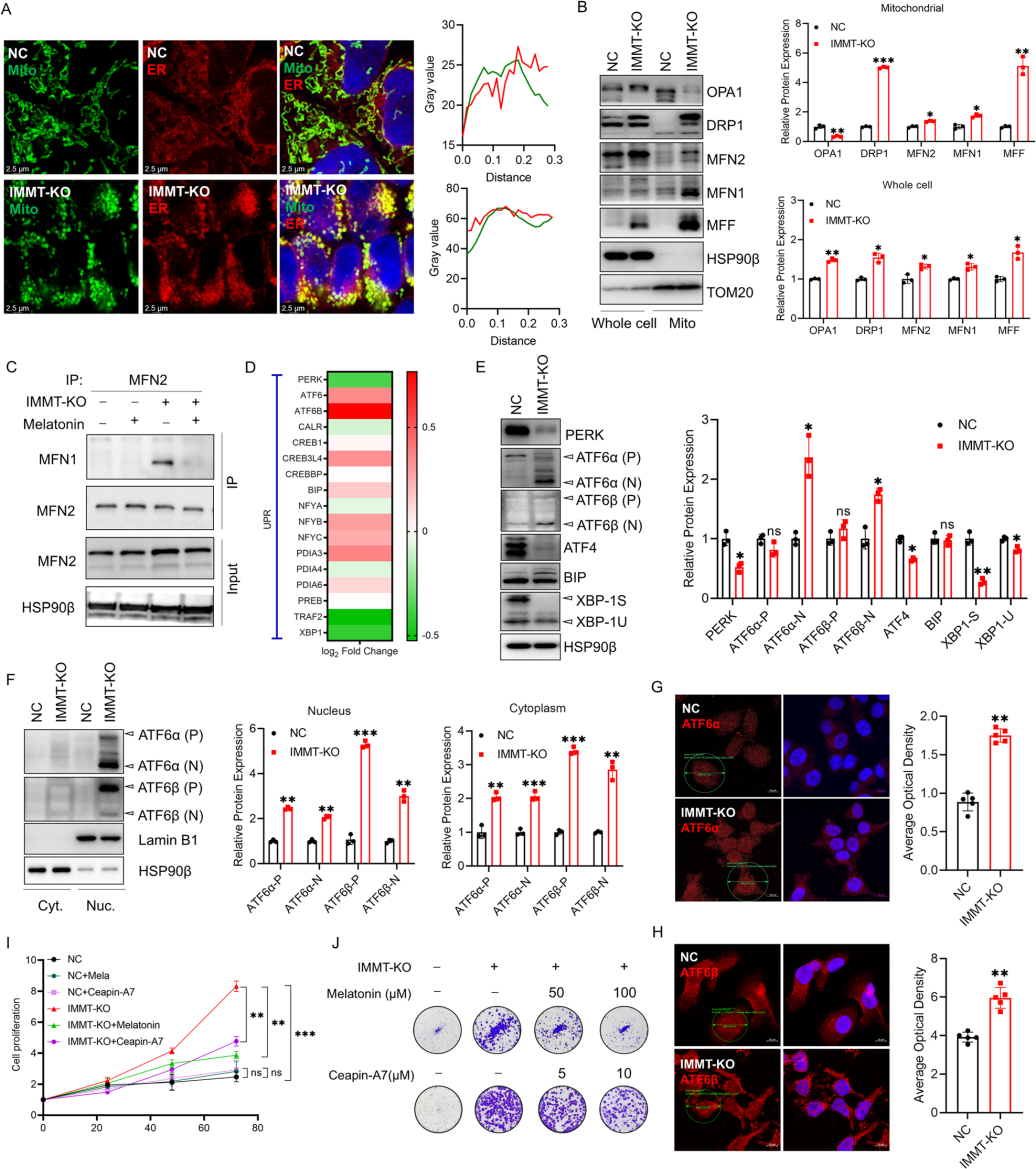
IMMT deficiency enhances ER-mitochondria interactions and activates stress response pathways. **A** Co-localization analysis of ER (ER-Tracker Red) and mitochondria (Mito-Tracker Green). **B** Quantitative analysis of mitochondrial dynamics-related proteins by subcellular fractionation combined with western blot. **C** IP assay examining MFN1-MFN2 protein interaction, with or without melatonin treatment (100 μM, 4 h). **D** Heatmap analysis of ER stress pathway-related molecules. **E** Western blot analysis of key ER stress marker proteins. **F** Nuclear/cytoplasmic fractionation assay quantifying ATF6α/β nuclear translocation. **G**, **H** IF assay detected subcellular localization of ATF6α/β in cells. (**I**) Cell proliferation measured by CCK-8 assay following treatment with melatonin (100 μM, 24 h) or Ceapin-A7 (10 μM, 24 h) (*n* = 5). **J** Colony formation assay following treatment with melatonin or Ceapin-A7 (*n* = 3). Data represent mean ± SD. Statistical significance: *P < 0.05, **P < 0.01, ***P < 0.001.

To further elucidate the upstream compensatory mechanisms following IMMT-KO, we performed GSEA using gene expression data from BC patients in the TCGA database and revealed significant enrichment of ERS-related signaling pathways in IMMT differentially expressed gene sets (Fig. S2A). Mitochondria-ER co-localization analysis revealed that IMMT-KO significantly increased contact frequency (Fig. 5A). This suggests IMMT deficiency triggers mitochondrial stress, subsequently inducing ER stress via inter-organelle crosstalk. Given the role of MAMs in regulating mitochondrial dynamics, we analyzed key regulatory proteins. Although mitochondrial dynamics regulators were generally upregulated in whole-cell lysates, mitochondrial fractionation demonstrated significantly reduced fusion protein OPA1 with concurrent elevation of fission proteins (DRP1, MFF) and fusion proteins (MFN1, MFN2) (Fig. 5B). Importantly, ER-localized MFN2 forms heterocomplexes with mitochondria-localized MFN1 to mediate inter-organelle tethering. IP experiments shown that IMMT-KO significantly enhanced the MFN1-MFN2 interaction (Fig. 5C), and treatment with the ER stress inhibitor melatonin abolished this enhanced interaction without altering protein expression levels (Figs. 5C and S3A), which further supports our hypothesis.

To further elucidate the mechanism by which IMMT-KO activates ERS, in-depth proteomic analysis and Western blot revealed significant upregulation of the ATF6 signaling pathway (Fig. 5D, E), which is considered a core regulatory molecule of ER stress. According to established mechanisms, activated ATF6 translocates from the ER to the Golgi apparatus, where it is cleaved by S1P/S2P proteases to generate an ~50-kDa active N-terminal fragment that enters the nucleus to exert transcriptional regulation [35]. In IMMT-KO cells, subcellular fractionation followed by immunoblotting revealed robust nuclear accumulation of the cleaved, transcriptionally active fragment of ATF6α and ATF6β (～50 kDa), consistent with canonical UPR activation (Fig. 5F). Notably, a strong signal corresponding to full-length ATF6β (～90 kDa) was also detected in the nuclear fraction—an observation that deviates from the classical model wherein only the processed N-terminal domain translocates to the nucleus. We propose that this likely reflects stress-induced retention of full-length ATF6β in perinuclear ER or nuclear envelope domains, which co-purify with nuclear fractions due to the continuity between the outer nuclear membrane and the ER. Given that IMMT loss disrupts MAMs and induces profound organelle stress, it is plausible that ER architecture remodeling alters ATF6β processing kinetics or subcellular distribution. Importantly, its nuclear enrichment was independently confirmed by IF (Fig. 5G, H).

To elucidate the biological functions of ATF6α/β, we treated IMMT-KO cells with Ceapin-A7 and melatonin, observing significantly impaired proliferative capacity (Fig. 5I, J), synchronized downregulation of PPARγ and FABP5/GPX8 (Fig. S3B), along with characteristic mitochondrial dysfunction including mtROS accumulation (Fig. S3C) and membrane potential depolarization (Fig. S3D). These findings reveal for the first time that IMMT deficiency coordinates inter-organelle communication and metabolic reprogramming through the ATF6-PPARγ axis, providing a theoretical foundation for targeted intervention against adaptive stress responses in tumor cells.

### TP53 mutation status associates with stress response heterogeneity to IMMT-KO

**Fig. 6:**
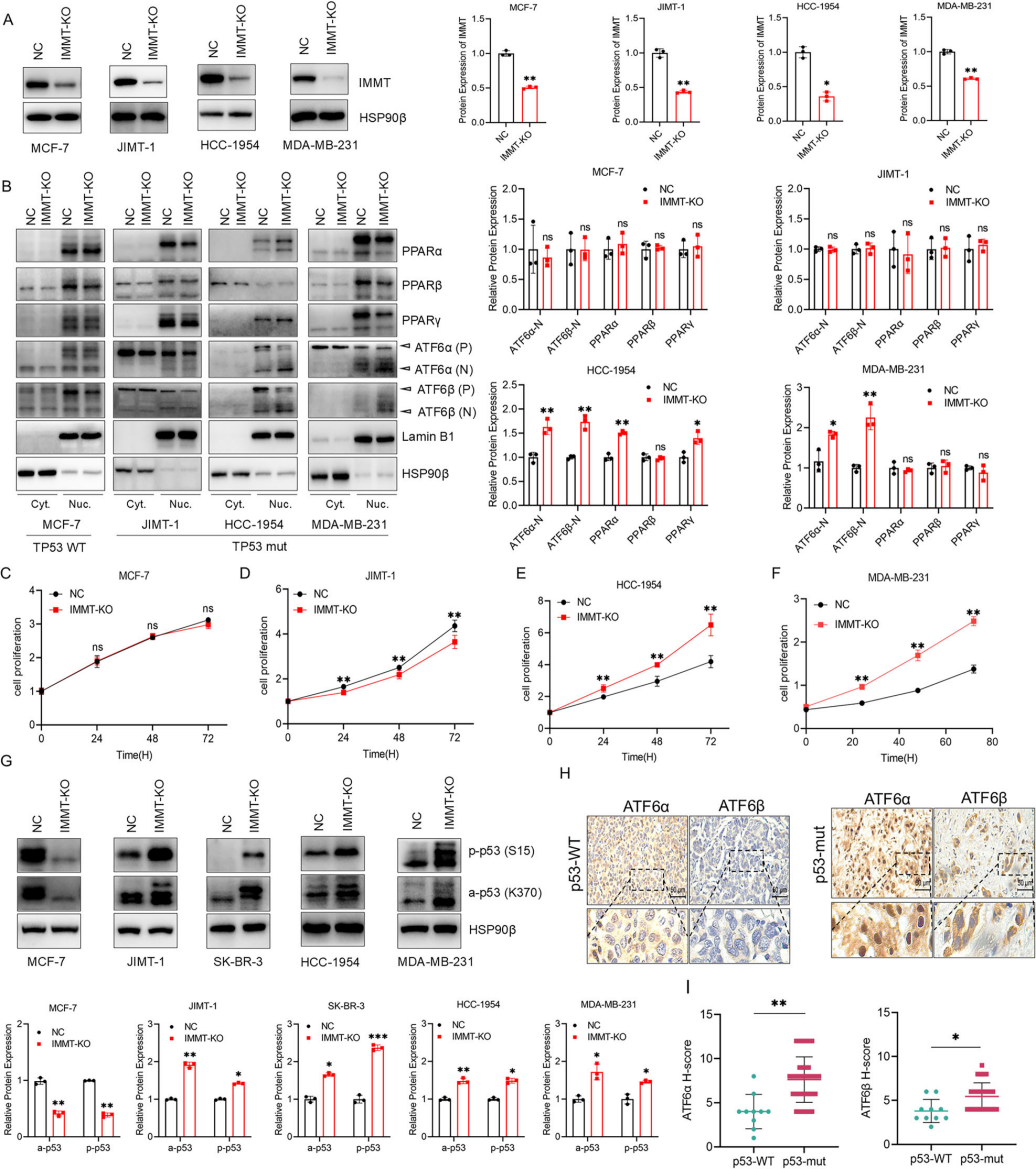
IMMT deficiency activates the ATF6-PPARγ axis in TP53-mutated BC. **A** Validation of IMMT-KO efficient in MCF-7, JIMT-1, HCC1954, and MDA-MB-231. **B** Nuclear/cytoplasmic fractionation assays were employed to analyze the nuclear translocation of PPAR and ATF6 subunits in the aforementioned cells following IMMT-KO. **C**–**F** CCK-8 assay was used to evaluate the proliferative activity of MCF-7, JIMT-1, HCC1954, and MDA-MB-231 cells following IMMT-KO (*n* = 5). **G** Western blot analysis was used to examine the expression of *p*-p53 (Ser15) and *a*-p53 (Lys370) in BC cells. **H** Representative IHC staining images of ATF6α and ATF6β in metastatic BC patient specimens. **I** H-score analysis was used to quantify IHC staining of ATF6α/β in recurrent metastatic tumor tissues with wild-type and mutant TP53. Data are presented as mean ± SD. *P < 0.05, **P < 0.01.

Given the heterogeneity of breast cancer, we next asked whether the pro-proliferative effect of IMMT-KO is universal or context-dependent. We therefore examined a panel of cell lines spanning major molecular subtypes and TP53 statuses: wild-type (MCF-7) and mutant (JIMT-1, MDA-MB-231, HCC1954, SK-BR-3) (Fig. 6A). We first observed that IMMT-KO robustly activated the ATF6 pathway and enhanced proliferation in two TP53-mutant cell lines: HCC-1954 and MDA-MB-231 (Fig. 6B, E, F). Notably, while both exhibited ATF6 activation, only HCC-1954 showed concomitant upregulation of PPARγ and PPARα, whereas MDA-MB-231 proliferated without PPARγ induction—suggesting context-dependent utilization of downstream effectors (Fig. 6B), It was further verified by in vivo experiments (Fig. S4A–D). In contrast, the TP53-wild-type MCF-7 cells failed to activate ATF6 or PPARγ and showed no proliferative advantage upon IMMT-KO (Fig. 6B, C), consistent with a requirement for altered p53 function. Strikingly, JIMT-1, a TP53 R248W mutant, HER2-amplified line closely related to SK-BR-3, failed to activate ATF6, PPARγ, or proliferate in response to IMMT-KO (Fig. 6B, D).

We assessed the levels of p53 phosphorylation (*p*-p53 S15) and acetylation (*a*-p53 K370) found that both modifications were markedly elevated in all TP53-mutant cell lines upon IMMT-KO (Fig. 6G). Moreover, *p*-p53 S15 and *a*-p53 K370 were markedly reduced in MCF-7 upon IMMT-KO, consistent with degradation of wild-type p53 under prolonged stress (Fig. 6G). Crucially, JIMT-1 exhibited strong induction of *p*-p53 S15 and *a*-p53 K370, comparable to other TP53-mutant lines (Fig. 6G). This key observation demonstrates that stress-induced post-translational modification and stabilization of mutant p53, while necessary, are not sufficient to drive the ATF6-mediated adaptive program. Instead, additional molecular determinants likely cooperate with modified mutant p53 to license ATF6 activation and metabolic rewiring.

To elucidate the impact of ATF6 signaling activation on BC patient prognosis, Kaplan-Meier survival analysis revealed that high expression of ATF6α/β was associated with shorter recurrence-free survival in TP53-mut patients (Fig. S4E–H). This association was further supported by IHC analysis of an independent cohort of 58 patients with recurrent/metastatic BC treated with systemic therapy, in which tumors harboring TP53 mutations exhibited significantly higher ATF6α/β protein levels and greater nuclear accumulation than TP53-wild-type tumors (Fig. 6H, I). These results reveal a strong clinical association between TP53 mutations and elevated ATF6α/β expression in BC, and further demonstrate that high ATF6α/β levels specifically predict poor prognosis among TP53-mutant patients. Together, these findings highlight ATF6 as a potential biomarker for risk stratification and a therapeutic vulnerability in TP53-mutant BC.

To directly assess whether TP53 loss is sufficient to drive ATF6 activation and proliferation, we performed TP53-KD experiments. The results revealed that TP53-KD did not significantly alter ATF6α protein levels, while ATF6β was only slightly upregulated (Fig. S4F). Notably, TP53-KD significantly suppressed cell proliferation (Fig. S4E). These findings indicate that the oncogenic effects of IMMT deficiency appear to depend on a pre-existing TP53 mutant background. Collectively, the oncogenic effect of IMMT deficiency appears to be contingent upon the pre-existing TP53-mutant background, which may provide a permissive landscape for metabolic rewiring and stress adaptation.

### ATF6α rewires stress signaling by heterodimerization with ATF6β upon IMMT-KO

**Fig. 7:**
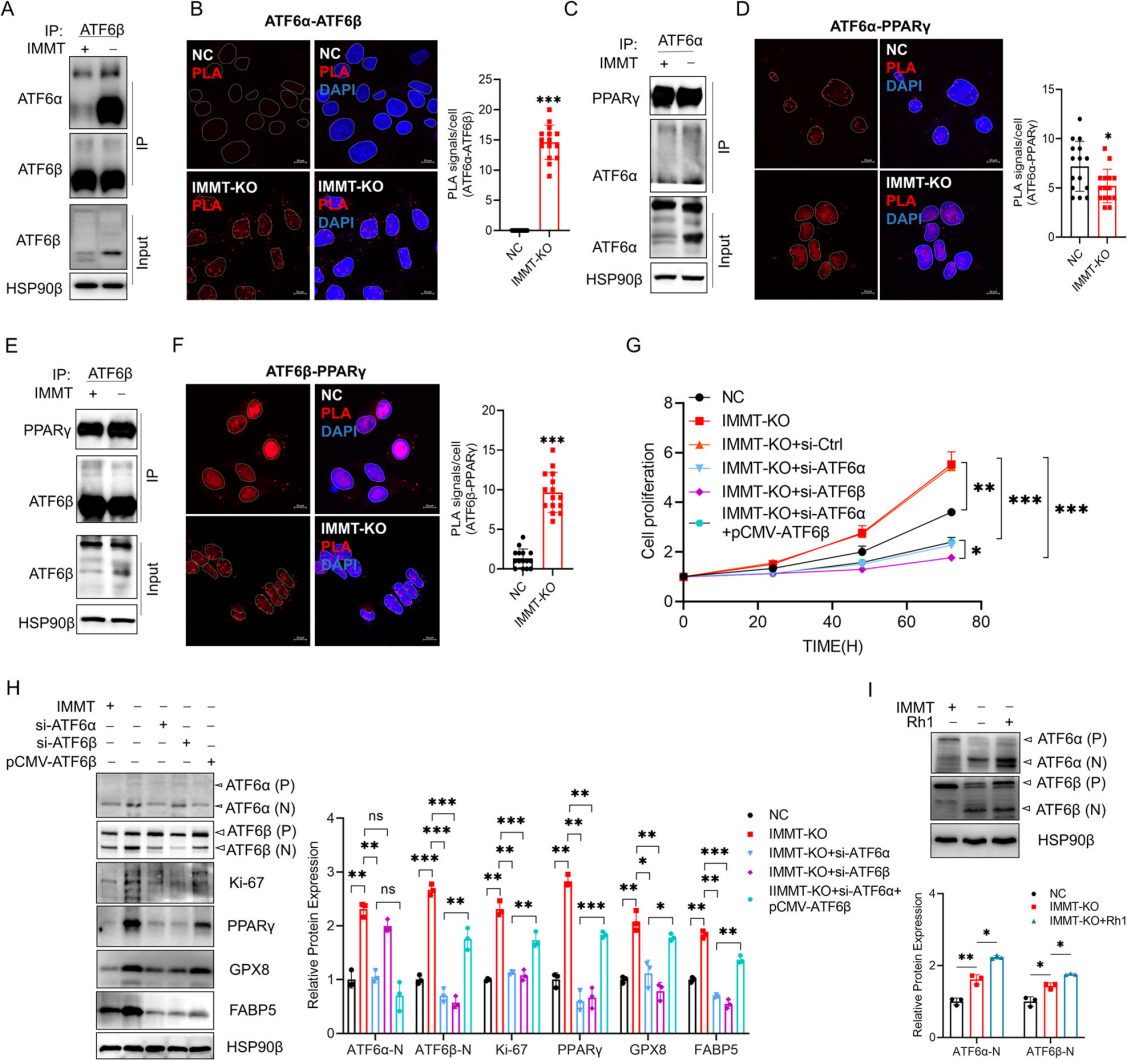
ATF6α/β interaction regulates the PPARγ-FABP5 axis in response to IMMT deficiency. **A** IP assay examining ATF6α-β protein interaction. **B** PLA analysis ATF6α-β interaction. **C** IP assay examining ATF6α-PPARγ protein interaction. **D** PLA analysis ATF6α-PPARγ interaction. **E** IP assay examining ATF6β-PPARγ protein interaction. **F** PLA analysis ATF6β-PPARγ interaction. **G** CCK-8 proliferation assay of SK-BR-3 cells transfected with siCtrl, siATF6α, siATF6β, or siATF6α + pMCV-ATF6β (*n* = 5). **H** Western blot and quantification of ATF6α, ATF6β, PPARγ, FABP5, and GPX8 in SK-BR-3 cells treated with siATF6α, siATF6β, or siATF6α + pMCV-ATF6β. **I** Western blot analysis of ATF6α and ATF6β expression after Rh1 treatment (10 μM, 4 h). Data represent mean ± SD. Statistical significance: *P < 0.05, **P < 0.01, ***P < 0.001.

Based on previous studies suggesting that ATF6β and PPARγ may function as restrictive factors for ATF6α (by binding to and inhibiting its excessive activation to prevent apoptosis) [36, 37], we systematically analyzed the interaction network of ATF6 isoforms under IMMT-KO conditions. IP experiments revealed that IMMT-KO significantly enhanced the formation of ATF6α/β heterodimers, particularly their active cleaved fragments (Fig. 7A). PLA experiments confirmed a significant increase in nuclear fluorescence foci in the IMMT-KO group compared to the control group (Fig. 7B), indicating enhanced nuclear interaction between ATF6α and ATF6β upon IMMT loss. Further detection of the interaction between PPARγ and the ATF6 subtype revealed that although both approaches revealed that PPARγ constitutively interacts with both ATF6α and ATF6β under basal conditions. However, a striking isoform-specific rewiring of this interaction occurred: the association between PPARγ and ATF6α was reduced (Fig. 7C, D), whereas binding between PPARγ and ATF6β was significantly enhanced (Fig. 7E, F).

To clarify the functional relationship between ATF6 isoforms, we performed isoform-specific knockdown followed by rescue experiments. Cell proliferation assays revealed that knockdown of either ATF6α or ATF6β significantly impaired cellular proliferative capacity (Fig. 7G). Notably, re-expression of ATF6β partially restored proliferation, underscoring its functional centrality in this pathway (Fig. 7G). At the molecular level, knockdown of ATF6α markedly reduced ATF6β protein levels, accompanied by downregulation of PPARγ, its downstream targets FABP5 and GPX8, and the proliferation marker Ki-67 (Fig. 7H). In contrast, ATF6β knockdown suppressed PPARγ signaling and its downstream effectors without affecting ATF6α protein abundance (Fig. 7H). Consistent with these observations, ectopic expression of ATF6β in ATF6α-deficient cells not only restored PPARγ expression but also rescued cell viability and proliferative capacity, demonstrating that ATF6β is both necessary and sufficient to mediate the pro-proliferative output of this axis. In contrast, pharmacological inhibition of PPARγ with Rh1 led to a compensatory increase in both ATF6α and ATF6β (Fig. 7I), likely reflecting feedback amplification due to unresolved organelle stress.

Together, these results indicate that ATF6α is required for maintaining ATF6β protein stability, whereas ATF6β acts as the direct effector of PPARγ-dependent transcriptional activation and proliferation. This functional interdependence aligns with our interaction data showing that IMMT-KO induces ATF6α–ATF6β heterodimer formation and selectively enhances the ATF6β–PPARγ interaction.

### Inhibition of ATF6 suppresses tumor growth in vivo by disrupting the ATF6α/β-PPARγ Axis

**Fig. 8:**
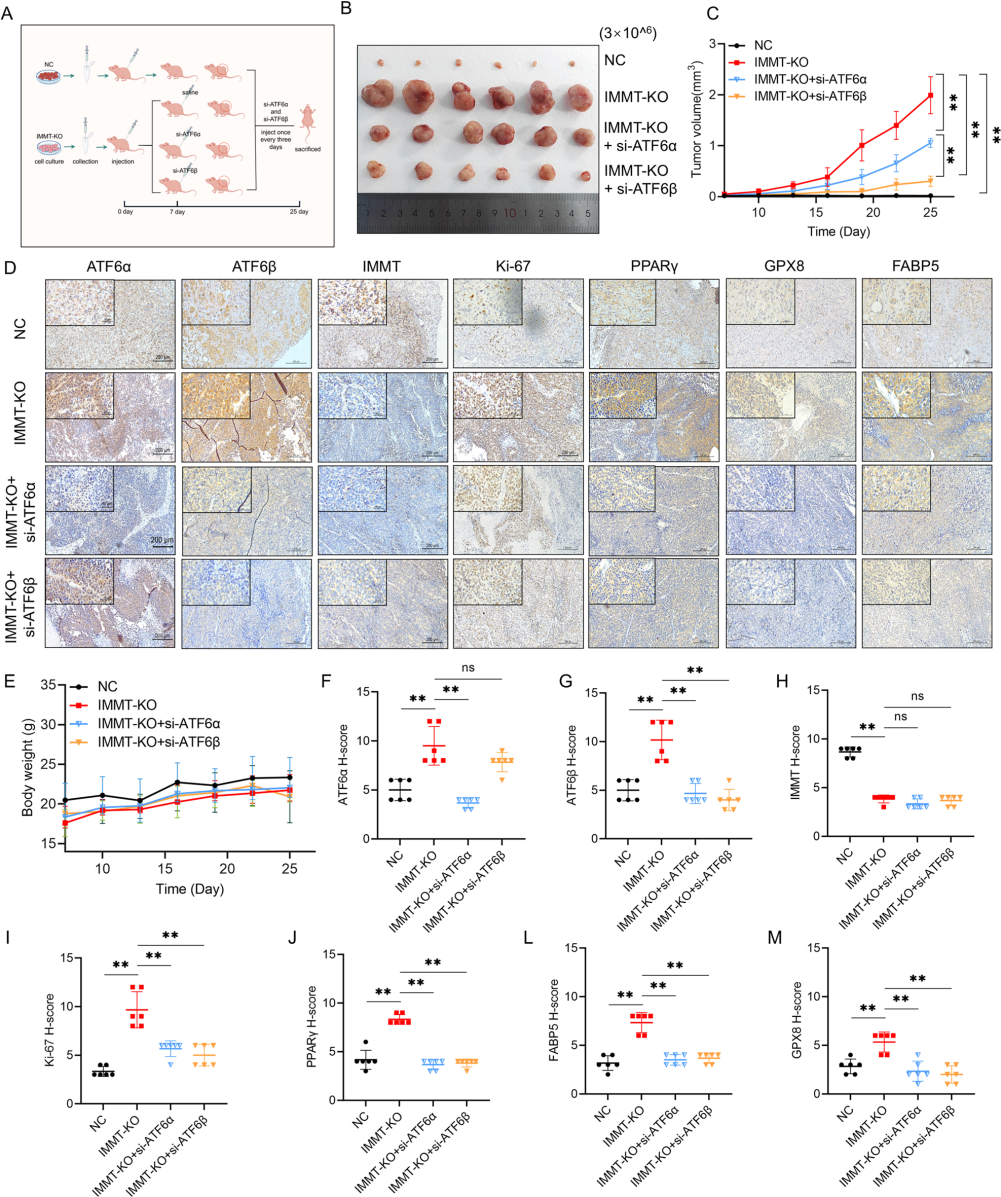
Therapeutic efficacy of targeting the ATF6-PPARγ axis in xenograft models. **A** A schematic diagram showing treatment strategies used in xenograft-bearing nude mice (By *Figdraw*). **B** Tumor images from BALB/c nude mice in different treatment groups (*n* = 6/group). **C** Tumor growth curve of tumor-bearing mice. **D** Representative IHC staining images of ATF6α, ATF6β, IMMT, Ki-67, PPARγ, GPX8 and FABP5 in dissected tumor tissues. **E** Body weight from tumor-bearing mice. **F**–**M** H-score analysis quantified IHC staining of aforementioned proteins in xenograft tumors. Data are presented as mean ± SD. Statistical significance: **P < 0.01.

To validate the functional significance of ATF6 isoforms in tumor growth, we established IMMT-KO xenograft models using SK-BR-3 cells and treated tumor-bearing mice with control siRNA, si-ATF6α, or si-ATF6β (*n* = 6 per group) (Fig. 8A). The results demonstrated that interference with either ATF6α or ATF6β significantly inhibited tumor proliferation, with the si-ATF6β treatment group showing markedly stronger tumor growth suppression compared to the si-ATF6α group, while no significant changes in body weight were observed across groups (Fig. 8B, C, E). IHC analysis confirmed significant reduction of IMMT expression in experimental groups, indicating successful model establishment (Fig. 8D). Concurrently, both si-ATF6α and si-ATF6β groups exhibited reduced numbers of Ki-67-positive cells and downregulation of key PPARγ signaling components—including PPARγ, FABP5, and GPX8 (Fig. 8D, H–M). Notably, knockdown of ATF6α markedly decreased ATF6β protein levels, whereas ATF6β depletion had no appreciable effect on ATF6α expression (Fig. 8D, F, G), recapitulating the asymmetric interdependence observed in vitro.

These findings collectively underscore that the ATF6α/β–PPARγ axis is essential for sustaining tumor cell proliferation and survival in vivo, and further highlight ATF6β as the pivotal effector within this pathway. Given its dual role in stabilizing the stress-adaptive response and directly engaging PPARγ, ATF6β represents a compelling node for therapeutic intervention in TP53-mutant BC.

## Discussion

Although mitochondrial dysfunction is a hallmark of cancer [38], how tumor cells convert chronic organelle stress into a pro-survival signal remains poorly understood. Here, we reveal that stable loss of the inner mitochondrial membrane protein IMMT triggers a stress-adaptive rewiring of the ATF6 branch of the UPR, a process that is strictly dependent on a pre-existing TP53-mutant background. Critically, we demonstrate that this adaptation is not mediated by canonical ATF6 signaling, but rather by a non-canonical ATF6 isoform complex: IMMT deficiency promotes ATF6α-ATF6β heterodimer formation, in which ATF6α acts as a stabilizing scaffold for ATF6β, enabling ATF6β to selectively engage PPARγ and drive metabolic reprogramming. This work thus redefines ATF6 not as a monolithic transcription factor, but as a plastic signaling module whose output is determined by isoform composition and mutational context.

Neoplastic cells characteristically employ metabolic reprogramming as a survival strategy against microenvironmental stress, with mitochondrial bioenergetics serving as a critical determinant of cellular fate decisions [39]. Consistent with Lin et al.‘s [24] report, our prior work established that transient IMMT silencing via siRNA-mediated knockdown suppresses BC cell proliferation through acute oxidative burst and OXPHOS collapse [23]. In contrast, the current study reveals that chronic IMMT depletion paradoxically promotes BC cell proliferation via adaptive metabolic reprogramming. Metabolic flux analysis demonstrated that, unlike transient knockdown, IMMT-KO cells maintain sufficient OXPHOS capacity. Notably, IMMT-KO triggers significant GSH accumulation coupled with a reduced NADP+/NADPH ratio. Mechanistically, IMMT-KO induces mitochondrial fragility, reducing mtROS buffering capacity and compelling cellular adaptation through two key strategies: expansion of antioxidant reserves and optimization of NADPH compartmentalization. These adaptations collectively sustain TCA cycle flux and OXPHOS efficiency at lower mtROS levels. These findings align with prior research by Pan et al. [40], which demonstrated distinct effects of transient knockdown versus stable knockout of the mitochondrial fusion gene OPA1 on stem cell pluripotency. These findings align with emerging evidence that stable genetic ablation, not transient perturbation, unmasks compensatory networks that support long-term survival [41]. Thus, the cellular response to mitochondrial dysfunction is temporally biphasic: an initial crisis phase followed by a rewired adaptive state.

The UPR can drive extensive metabolic reprogramming through its regulation of MAMs, yet how specific UPR branches interface with metabolic regulators remains unclear [22, 42, 43]. We find that chronic IMMT loss selectively activates the ATF6 arm, leading to PPARγ upregulation and enhanced PPARγ–FABP5 interaction, which together restore mitochondrial membrane potential and alleviate ER stress. Importantly, this is not a generic UPR response: ATF6β, not ATF6α, directly binds PPARγ, forming a functional transcriptional complex that drives expression of antioxidant (GPX8) and lipid metabolism (FABP5) genes. These results are consistent with previous findings reported by Cao’s [44] team. This regulatory axis likely represents a critical adaptive strategy through which tumor cells enhance survival by coordinately upregulating lipid metabolism and antioxidant capacity. Notably, in our IMMT-KO model, the activation of the UPR did not lead to a significant increase BIP expression. This may be attributed to the concurrent compensatory activation of autophagy or antioxidant pathways [45, 46]. Specifically, mitophagy triggered by IMMT-KO was observed in our additional experiments (data not shown).

Our unexpected findings reveal a potential correlation between TP53 mutation status and ATF6 activation. While wild-type p53 undergoes rapid MDM2-mediated ubiquitination and degradation to maintain homeostasis, TP53 mutations lead to structural alterations that prevent degradation, resulting in mutant p53 accumulation and subsequent cancer progression [47, 48]. Intriguingly, our results demonstrate that IMMT-KO induces robust ATF6 activation in lines such as HCC1954 and MDA-MB-231, it fails to do so in the JIMT-1 cells, despite their HER2 amplification and expression of mutant p53. Notably, although JIMT-1 exhibits stress-induced phosphorylation (S15) and acetylation (K370) of p53 similar to responsive lines, it does not engage the ATF6–PPARγ axis. This indicates that the mere presence of mutant p53 and oncogenic drivers is insufficient. An emerging concept in cancer biology is that certain TP53 mutations confer gain-of-function properties that enhance cellular fitness under stress [49, 50]. Our data support a model in which mutant p53 does not directly activate ATF6, but rather creates a permissive cellular environment that enables survival through the acute phase of mitochondrial and ER stress induced by IMMT loss. In TP53 wild-type cells, such stress would likely trigger p53-dependent cell cycle arrest or apoptosis, thereby preventing adaptive metabolic reprogramming. In contrast, TP53-mutant cell may endure this crisis phase, allowing the ATF6β–PPARγ axis to orchestrate a pro-proliferative transcriptional program. This “stress resilience” conferred by TP53 mutation could explain the striking context dependency of the IMMT-KO phenotype and underscores the importance of genetic background in determining cellular responses to organelle dysfunction.

Our work fundamentally revises the prevailing view of ATF6 isoforms. Contrary to the notion that ATF6β functions primarily as a dominant-negative inhibitor of ATF6α [51], we demonstrate that ATF6α and ATF6β cooperate as a heterodimeric unit under chronic stress. ATF6α stabilizes ATF6β protein levels, likely through direct interaction, while ATF6β serves as the selective transcriptional partner for PPARγ. Depletion of either isoform disrupts the axis: ATF6α loss destabilizes ATF6β, whereas ATF6β loss abrogates PPARγ engagement. Rescue experiments confirm that ATF6β is both necessary and sufficient to restore proliferation in ATF6α-deficient cells. Thus, the two isoforms exhibit functional interdependence, not antagonism, revealing a previously unappreciated layer of UPR regulation through isoform-specific complex assembly.

The shift from acute stress sensitivity to chronic adaptation implies a critical window during which only a subset of cells successfully rewire their stress response [52, 53]. Based on our data, we propose that the initial trigger is not sustained mtROS elevation but rather the structural and bioenergetic instability caused by IMMT loss, including mitochondrial fragmentation and transient membrane potential fluctuations. Intriguingly, we observed enhanced MFN1–MFN2 interaction in IMMT-KO cells, suggesting reinforced mitochondria–ER tethering via MAMs, which may facilitate ATF6 activation by promoting ER stress signal propagation. Because stable IMMT-KO models are derived from clonal selection, they inherently represent the endpoint of successful adaptation. The exact sequence of molecular events, particularly whether ATF6 activation precedes or follows metabolic rewiring, remains unclear. Future studies employing inducible IMMT knockout coupled with high-resolution time-course metabolomics, phosphoproteomics, and live imaging of organelle dynamics will be essential to dissect the causal hierarchy of this adaptive cascade.

In conclusion, our work uncovers a novel compensatory pathway activated by mitochondrial dysfunction and redefines the role of ATF6β. More importantly, we identify that this pathway’s activation is contingent upon TP53 mutation status, thereby presenting a novel biomarker for patient stratification and a therapeutic vulnerability for the challenging subset of TP53-mutant BCs.

## Supplementary information

Supplement Figure 1

Supplement Figure 2

Supplement Figure 3

Supplement Figure 4

Supplementary Figure Legends

Supplementary Table 1

Supplementary Table 2

Supplementary Table 3

## Data Availability

The datasets supporting this study are available within the article and from the corresponding author upon request.
