# Supplementary figures and images for: Metabolic adaptation to IMMT deficiency through the ATF6-PPARγ axis is contingent on TP53 mutation status in breast cancer
Source: Cell Death Dis. 2026 Apr 28;17(1):565. doi: 10.1038/s41419-026-08813-y (PMC13261075; doi:10.1038/s41419-026-08813-y)

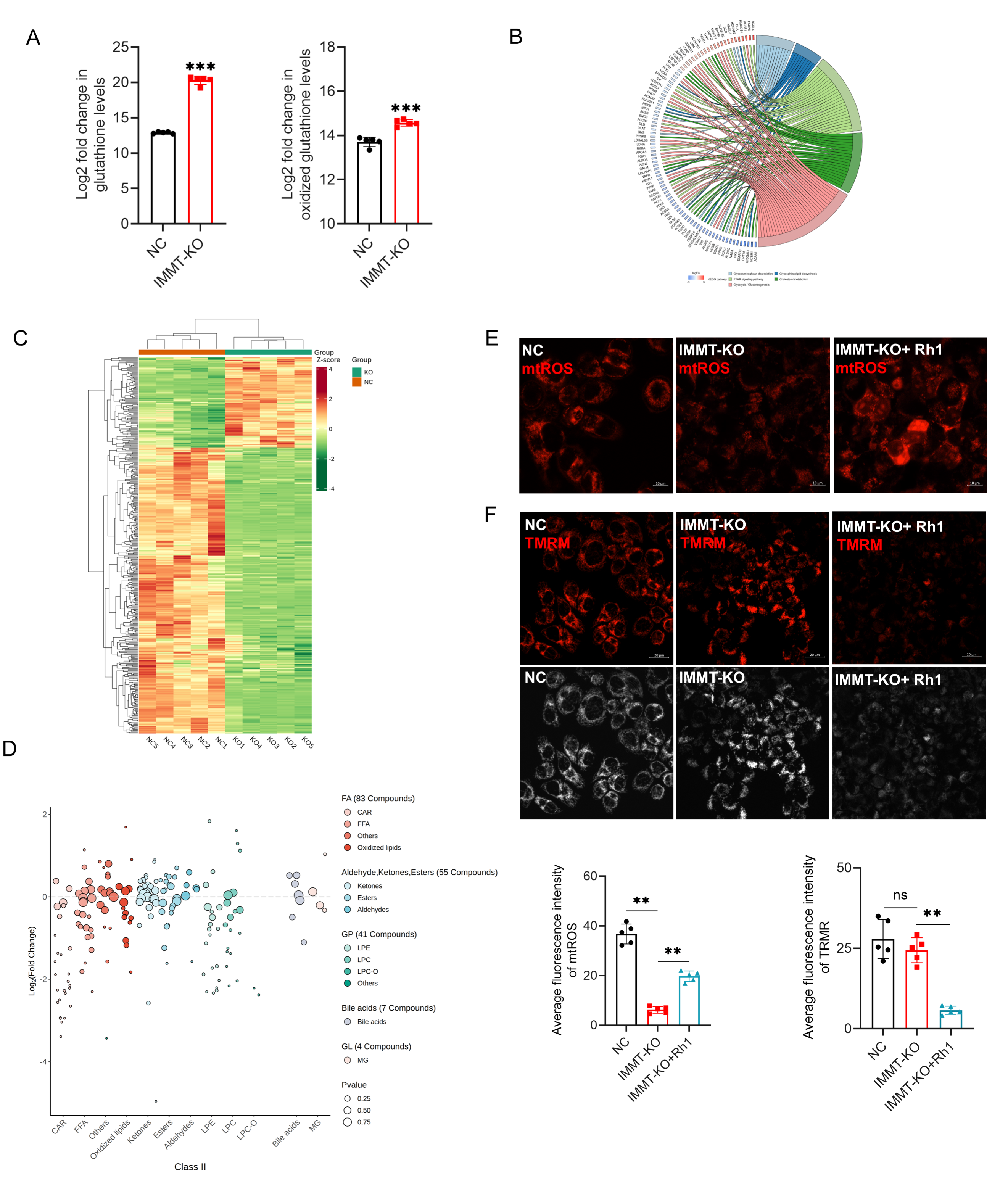

Supplement: Supplementary file 1 — Supplement Figure 1 [file 41419_2026_8813_MOESM1_ESM.tif]

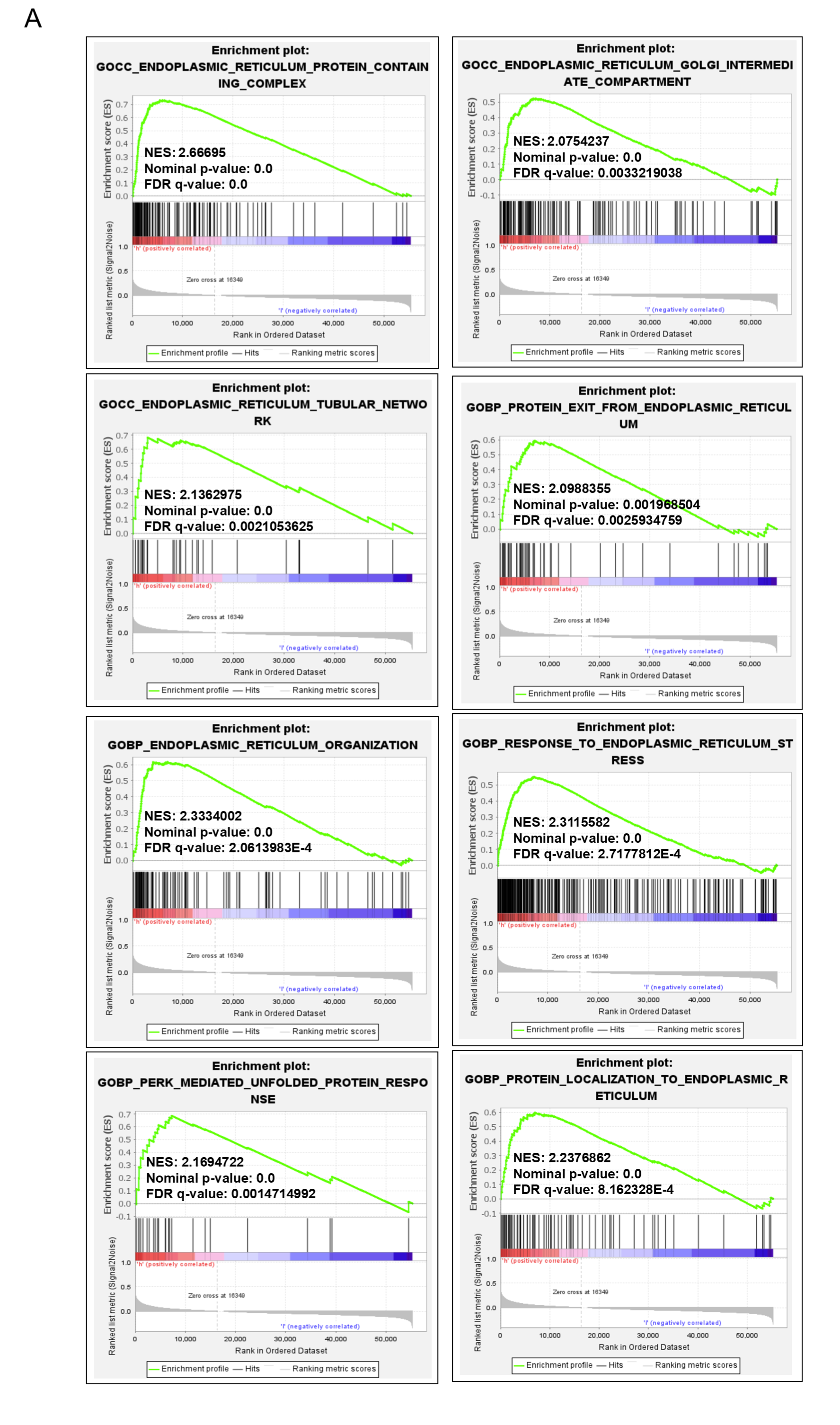

Supplement: Supplementary file 2 — Supplement Figure 2 [file 41419_2026_8813_MOESM2_ESM.tif]

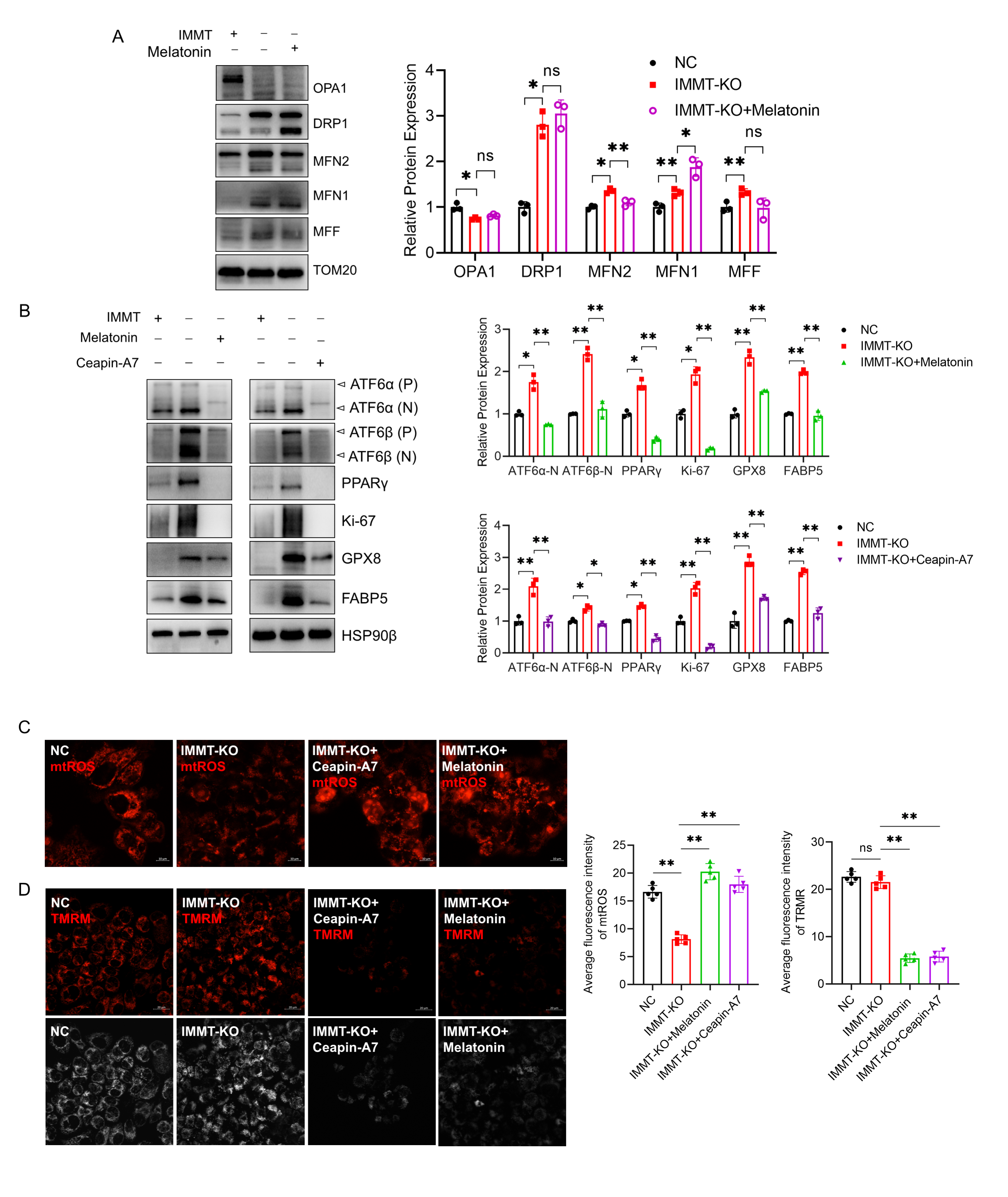

Supplement: Supplementary file 3 — Supplement Figure 3 [file 41419_2026_8813_MOESM3_ESM.tif]

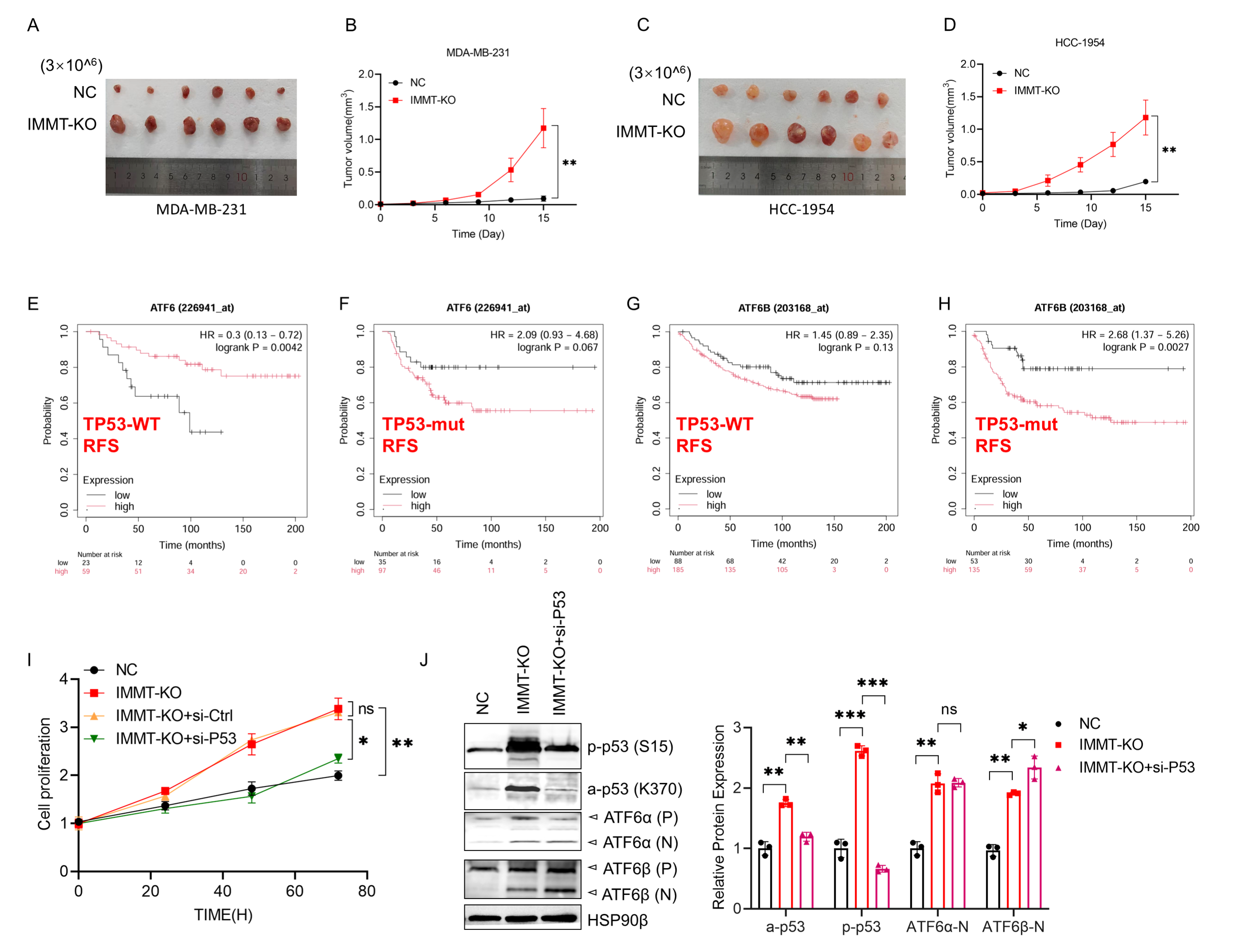

Supplement: Supplementary file 4 — Supplement Figure 4 [file 41419_2026_8813_MOESM4_ESM.tif]
