# Supplementary material for: Metabolic adaptation to IMMT deficiency through the ATF6-PPARγ axis is contingent on TP53 mutation status in breast cancer
Source: Cell Death Dis. 2026 Apr 28;17(1):565. doi: 10.1038/s41419-026-08813-y (PMC13261075; doi:10.1038/s41419-026-08813-y)
Supplement: Supplementary file 5 — Supplementary Figure Legends [file 41419_2026_8813_MOESM5_ESM.docx]

**SUPPLEMENTAL FIGURE LEGENDS**

**Figure S1.** **A.** IMMT-KO increases both reduced (GSH) and oxidized (GSSG) glutathione levels. **B.** Chord diagram illustrating interactions between significantly enriched pathways and their core proteins. **C.** Heatmap showing the relative levels of metabolites in IMMT-KO compared to NC cells. **D.** Bubble plot illustrating changes in lipid metabolites between IMMT-KO and NC cells. **E.** Mitochondrial superoxide levels measured by SOX™ Red fluorescence. **F.**  Mitochondrial membrane potential (ΔΨm) was evaluated using TMRM fluorescence staining. Data represent mean ± SD. Statistical significance: ***P* < 0.01, ****P* < 0.001.

**Figure S2.** **A.** GSEA of BC-related pathways in IMMT-high versus IMMT-low expression groups (TCGA cohort).

**Figure S3. A.** Quantitative analysis of mitochondrial dynamics-related proteins after melatonin treatment by Western blot. **B.** Western blot analysis of ATF6α, ATF6β, PPARγ, Ki-67, GPX8, and FABP5 expression in SK-BR-3 cells treated with melatonin or Ceapin-A7. **C.** Mitochondrial superoxide levels measured by SOX™ Red fluorescence. **D.** Mitochondrial membrane potential (ΔΨm) was evaluated using TMRM fluorescence staining. Data represent mean ± SD. Statistical significance:  **P* < 0.05, ***P* < 0.01.

**Figure S4.** **A-B.** Representative photographs of excised tumors from control and IMMT-KO MDA-MB-231 xenografts at the experimental endpoint (n=6). **C-D.** Representative photographs of excised tumors from control and IMMT-KO HCC1954 xenografts at the experimental endpoint (n=6). **E-H.** Kaplan-Meier survival curves were used to analyze the relationship between ATF6 subunit expression and relapse-free survival in BC patients with wild-type and mutated TP53. **I.** Cell proliferation assay following TP53 knockdown. **J.** Western blot analysis the expressions of p-P53, a-P53, ATF6α and ATF6β after knockdown P53. Data are presented as mean ± SD. Statistical significance: **P* < 0.05, ***P* < 0.01, ****P* < 0.001.
