# Supplementary material for: Metabolic adaptation to IMMT deficiency through the ATF6-PPARγ axis is contingent on TP53 mutation status in breast cancer
Source: Cell Death Dis. 2026 Apr 28;17(1):565. doi: 10.1038/s41419-026-08813-y (PMC13261075; doi:10.1038/s41419-026-08813-y)
Supplement: Supplementary file 6 — Supplementary Table 1 [file 41419_2026_8813_MOESM6_ESM.docx]

**Supplementary Table 1.** TP53 status in human cell lines used in this work is summarized in the following table:

| Cell line | Species | Tissue | p53 status | Protein Change | Other key driver mutations/deletions |
| --- | --- | --- | --- | --- | --- |
| MDA-MB 231 | human | Breast | missense | p.R280K | KRAS G13D, PTEN loss |
| SK-BR-3 | human | Breast | missense | p.R175H | ERBB2 amplification, PIK3CA WT, PTEN WT |
| HCC-1954 | human | Breast | missense | p.Y163C | ERBB2 amplification, PIK3CA H1047R, PTEN loss |
| JIMT-1 | human | Breast | missense | p.R248W | ERBB2 amplification, PTEN loss, PIK3CA WT |
| MCF-7 | human | Breast | wild-type | --- | PIK3CA H1047R, GATA3 mut, ESR1+, PTEN WT |
