# Supplementary material for: Metabolic adaptation to IMMT deficiency through the ATF6-PPARγ axis is contingent on TP53 mutation status in breast cancer
Source: Cell Death Dis. 2026 Apr 28;17(1):565. doi: 10.1038/s41419-026-08813-y (PMC13261075; doi:10.1038/s41419-026-08813-y)
Supplement: Supplementary file 7 — Supplementary Table 2 [file 41419_2026_8813_MOESM7_ESM.docx]

**Supplementary Table 2.** Primary antibodies used are listed in the following table:

| **Antibody** | **Vendor (catalogue n.)** |
| --- | --- |
| Anti-ATF6α | Abclonal (A0202) |
| Anti-ATF6β | Proteintech (15794-1-AP) |
| Anti-IMMT | Proteintech (68226-1-Ig) |
| Anti-Ki-67 | Proteintech (84192-4-RR) |
| Anti-PCNA | HUABIO (ET1605-38) |
| Anti-HSP90β | HUABIO (ET1605-56) |
| Anti-HK-1 | Cell Signaling Technology (#2024) |
| Anti-HK-2 | Cell Signaling Technology (#2867) |
| Anti-PKM2 | Cell Signaling Technology (#4053) |
| Anti-LDHA | Cell Signaling Technology (#3582) |
| Anti-OXPHOS Cocktail | Proteintech (PK30006) |
| Anti-ACSL4 | Cell Signaling Technology (#38493) |
| Anti-GPX4 | Cell Signaling Technology (#59735) |
| Anti-GPX8 | Proteintech (85120-1-RR) |
| Anti-CD36 | Cell Signaling Technology (#28109) |
| Anti-FABP5 | Proteintech (66299-1-Ig) |
| Anti-SLC16A1 | Santa Cruz (sc-365501) |
| Anti-PPAR-α | Absin (abs124681) |
| Anti-PPAR-β | Santa Cruz ((sc-74517)) |
| Anti-PPAR-γ | HUABIO (ET1702-57) |
| Anti-Lamin B | HUABIO (ET1606-27) |
| Anti-PERK | Santa Cruz (sc-377400) |
| Anti-ATF4 | Santa Cruz (sc-390063) |
| Anti-BIP | Proteintech (66574-1-Ig) |
| Anti-XBP-1 | Santa Cruz (sc-8015) |
| Anti-OPA1 | Abcam (ab157457) |
| Anti-MFN1 | Abcam (ab221661) |
| Anti-MFN2 | Abcam (ab124773) |
| Anti-DRP1 | Abcam (ab184247) |
| Anti-MFF | Abcam (ab129075) |
| Anti-TOM20 | Abclonal (A19403) |
| Anti-p-p53 | Abcam (ab223868) |
| Anti-a-p53 | Abcam (ab183544) |
| Multi-rAb™ CoraLite® Plus 488-Goat Anti-Mouse Recombinant Secondary Antibody (H+L) | Proteintech (RGAM002) |
| Multi-rAb™ CoraLite® Plus 594-Goat Anti-Rabbit Recombinant Secondary Antibody (H+L) | Proteintech (RGAR004) |
| HRP Conjugated Goat anti-Rabbit IgG polyclonal Antibody | HUABIO (HA1001) |
| HRP Conjugated Goat anti-Mouse IgG polyclonal Antibody | HUABIO (HA1006) |
