# Supplementary material for: Metabolic adaptation to IMMT deficiency through the ATF6-PPARγ axis is contingent on TP53 mutation status in breast cancer
Source: Cell Death Dis. 2026 Apr 28;17(1):565. doi: 10.1038/s41419-026-08813-y (PMC13261075; doi:10.1038/s41419-026-08813-y)
Supplement: Supplementary file 8 — Supplementary Table 3 [file 41419_2026_8813_MOESM8_ESM.docx]

**Supplementary Table 3.** The clinicopathologic information of the patients.

| Characteristics | Number of cases |
| --- | --- |
| Age |  |
| <55 | 42 |
| ≥55 | 16 |
| Ki-67 (%) |  |
| <20 | 5 |
| ≥20 | 53 |
| ER status |  |
| Negative | 14 |
| Positive | 44 |
| PR status |  |
| Negative | 19 |
| Positive | 39 |
| HER-2 status |  |
| Negative | 43 |
| Positive | 15 |
| Prior treatments |  |
| Chemotherapy | 36 |
| Endocrine Therapy | 4 |
| Chemotherapy + Endocrine Therapy | 6 |
| Anti-HER2 Therapy | 9 |
| NO TREATMENT | 3 |
| Molecular subtypes |  |
| Luminal A | 13 |
| Luminal B | 32 |
| HER-2 | 3 |
| TNBC | 10 |
| P53 status |  |
| wild type | 10 |
| mutation | 48 |
| Puncture site |  |
| chest wall | 16 |
| supraclavicular lymph node | 15 |
| liver | 12 |
| lung | 7 |
| axillary lymph node | 2 |
| cervical lymph nodal | 2 |
| upper back | 1 |
| thoracic vertebrae | 1 |
| humerus | 1 |
| pleural | 1 |
